# Hypoxia-Inducible Factor-2α Is an Essential Catabolic Regulator of Inflammatory Rheumatoid Arthritis

**DOI:** 10.1371/journal.pbio.1001881

**Published:** 2014-06-10

**Authors:** Je-Hwang Ryu, Chang-Suk Chae, Ji-Sun Kwak, Hwanhee Oh, Youngnim Shin, Yun Hyun Huh, Choong-Gu Lee, Yong-Wook Park, Churl-Hong Chun, Young-Myeong Kim, Sin-Hyeog Im, Jang-Soo Chun

**Affiliations:** 1 Cell Dynamics Research Center and School of Life Sciences, Gwangju Institute of Science and Technology, Gwangju, Republic of Korea; 2 Research Center for Biomineralization Disorders and Dental Science Research Institute, School of Dentistry, Chonnam National University, Gwangju, Republic of Korea; 3 Department of Rheumatology, Chonnam National University Medical School and Hospital, Gwangju, Republic of Korea; 4 Department of Orthopedic Surgery, Wonkwang University School of Medicine, Iksan, Republic of Korea; 5 Department of Molecular and Cellular Biochemistry, School of Medicine, Kangwon National University, Chuncheon, Republic of Korea; 6 Academy of Immunology and Microbiology, Institute for Basic Science, and Department of Integrative Biosciences and Biotechnology, Pohang University of Science and Technology, Pohang, Republic of Korea; National Jewish Medical and Research Center/Howard Hughes Medical Institute, United States of America

## Abstract

Rheumatoid arthritis (RA) is a systemic autoimmune disorder that manifests as chronic inflammation and joint tissue destruction. However, the etiology and pathogenesis of RA have not been fully elucidated. Here, we explored the role of the hypoxia-inducible factors (HIFs), HIF-1α (encoded by *HIF1A*) and HIF-2α (encoded by *EPAS1*). HIF-2α was markedly up-regulated in the intimal lining of RA synovium, whereas HIF-1α was detected in a few cells in the sublining and deep layer of RA synovium. Overexpression of HIF-2α in joint tissues caused an RA-like phenotype, whereas HIF-1α did not affect joint architecture. Moreover, a HIF-2α deficiency in mice blunted the development of experimental RA. HIF-2α was expressed mainly in fibroblast-like synoviocytes (FLS) of RA synovium and regulated their proliferation, expression of RANKL (receptor activator of nuclear factor–κB ligand) and various catabolic factors, and osteoclastogenic potential. Moreover, HIF-2α–dependent up-regulation of interleukin (IL)-6 in FLS stimulated differentiation of T_H_17 cells—crucial effectors of RA pathogenesis. Additionally, in the absence of IL-6 (*Il6* ^−/−^ mice), overexpression of HIF-2α in joint tissues did not cause an RA phenotype. Thus, our results collectively suggest that HIF-2α plays a pivotal role in the pathogenesis of RA by regulating FLS functions, independent of HIF-1α.

## Abstract 2

Hypoxia-inducible factor-2α (HIF-2α) is sufficient to cause experimental rheumatoid arthritis and acts to regulate the functions of fibroblast-like cells from tissue surrounding joints, independent of HIF-1α.

## Author Summary

Rheumatoid arthritis (RA) is a systemic autoimmune disorder characterized by chronic inflammation in joint tissues leading to destruction of cartilage and bone. Despite some therapeutic advances, the etiology of RA pathogenesis is not yet clear, and effective treatment of RA remains a significant, unmet medical need. Hypoxia is a prominent feature of inflamed tissue within RA-affected joints, and earlier work has implicated limited involvement of hypoxia-inducible factor (HIF)-1 α. We explored the role of a second HIF family member, HIF-2α, in RA pathogenesis. We showed that HIF-2α is markedly increased in the tissue lining the RA-affected joints. Notably and in contrast to HIF-1α, when overexpressed in normal mouse joint tissues, HIF-2α is sufficient to cause RA-like symptoms. Conversely, an HIF-2α deficiency blocks the development of experimental arthritis in mice. We discovered further that HIF-2α regulates RA pathogenesis by modulating various RA-associated functions of joint-specific fibroblast-like cells, including proliferation, expression of cytokines, chemokines, and matrix-degrading enzymes, and bone-remodeling potential. HIF-2α also increases the ability of these cells to promote interleukin-6–dependent differentiation of T_H_17 cells, a known effector of RA pathogenesis. We thus show that HIF-1α and HIF-2α have distinct roles and act via different mechanisms in RA pathogenesis.

## Introduction

Rheumatoid arthritis (RA) is a chronic inflammatory autoimmune disease that mainly targets the synovial membrane, resulting in destruction of the joint architecture. The pathophysiology of RA involves numerous cell types, including T cells, B cells, macrophages, synoviocytes, chondrocytes, and osteoclasts, all of which contribute to the process of RA pathogenesis [1]. T-cell–mediated autoimmune responses play an important role in RA pathogenesis, in which interleukin (IL)-17–producing T-helper cells (T_H_17) act as crucial effectors [1],[2]. RA is characterized by synovial hyperplasia and synovitis with infiltration of immune cells. Synovial tissues express numerous cytokines that have been directly implicated in many immune processes of RA pathogenesis [1],[3]. Additionally, an aggressive front of hyperplastic synovium, called the pannus, invades and destroys mineralized cartilage and bone through the action of osteoclasts [1],[3]. Synovial hyperplasia results from a marked increase in macrophage-like and fibroblast-like synoviocytes (FLS). Accumulating evidence indicates that activated FLS are among the key players in RA joint destruction [4]. FLS actively contribute to the initiation, propagation, and maintenance of synovial inflammation through secretion of factors and direct cell–cell interactions. For instance, cytokines and chemokines produced by FLS attract T cells to RA synovium, and the interaction of FLS with T cells results in activation of both cell types. FLS in the inflamed synovium also contribute to RA pathogenesis by producing matrix-degrading enzymes involved in cartilage destruction; RANKL (receptor activator of nuclear factor–κB ligand), which regulates osteoclast differentiation, leading to bone erosion; and angiogenic factors associated with blood vessel formation [4]. Despite therapeutic advances, the etiology of RA pathogenesis has not yet been entirely elucidated, and effective treatment of RA remains a significant unmet medical need.

A prominent feature of the inflamed RA synovium is hypoxia [5]–[7], suggesting a possible role for hypoxia-inducible factors (HIFs) in RA pathogenesis. HIFs are members of a transcription factor family that act as “master regulators” of the adaptive response to hypoxia [8],[9]. Of the three isoforms, HIF-1α (encoded by *HIF1A*) and HIF-2α (encoded by *EPAS1*) are the most extensively studied. HIF-1α is up-regulated in RA synovium [10]–[12], where it appears to be associated with angiogenesis [5]–[7]. HIF-1α is also expressed in T_H_17 cells, where it serves to regulate T_H_17/T_reg_ balance; a lack of HIF-1α in T_H_17 cells impairs their differentiation [13],[14]. Additionally, loss of HIF-1α in myeloid cells reduces the RA pathogenesis caused by K/BxN serum transfer [15]. Although these results suggest that HIF-1α is an important mediator of RA pathogenesis, whether HIF-1α is sufficient to cause RA pathogenesis *in vivo* has not been previously demonstrated. Most strikingly, HIF-2α, which is closely related to HIF-1α, has not yet been investigated for a role in RA pathogenesis. Indeed, despite many similarities between HIF-1α and HIF-2α, these two isoforms show different sensitivity to oxygen tension and display distinct, and sometimes opposing, cellular activities [8],[9]. Here, we present an extensive study of the function of HIF-2α in experimental inflammatory arthritis in mice. We also investigated whether the role of HIF-2α is independent of, complementary to, or redundant with that of HIF-1α in the development and pathogenesis of experimental RA. We report here that HIF-2α is an essential catabolic regulator of RA pathogenesis, independent of the action of HIF-1α.

## Results

### HIF-1α and HIF-2α Are Differentially Up-Regulated in RA Synovium

**Figure 1:**
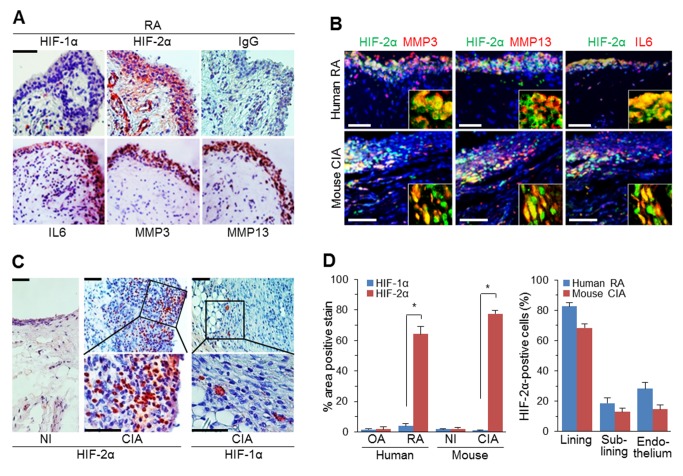
HIF-1α and HIF-2α are differentially up-regulated in RA synovium. (A) Representative images of human RA synovium immunostained for HIF-1α, HIF-2α, IL6, MMP3, and MMP13 (*n* = 10). (B) Representative images of human RA and mouse CIA synovial sections (*n* = 8) immunostained for HIF-2α and a RA synovium marker (MMP3, MMP13, or IL6) and counterstained with DAPI (triple stained). Insets are enlarged images of double-stained cells. (C) Representative images of HIF-1α and HIF-2α immunostaining in the knee synovia of CIA and NI control DBA/1J mice (*n* = 10). (D) Relative expression levels of HIF-1α and HIF-2α in synovial cells (left) (*n* = 10). HIF-2α–positive cells were counted in the indicated compartments of RA synovium (right) (*n* = 5). Values are means ± SEM (*p<0.0005). Scale bar, 50 µm.

To explore possible functions of HIFs in RA pathogenesis, we first examined the expression pattern of HIFs by immunostaining human RA joint sections. HIF-2α was highly expressed in the intimal lining of human RA synovium, where other markers of inflamed RA synovium were expressed, including IL-6, matrix metalloproteinase (MMP)3, and MMP13 (Figure 1A). Indeed, double immunostaining for HIF-2α and these markers revealed their co-localization in human RA synovium (Figure 1B). HIF-2α was also up-regulated in tartrate-resistant acid phosphatase (TRAP)-positive osteoclasts in bone tissue and chondrocytes in damaged cartilage, but not in the intact, undamaged part of human RA cartilage (Figure S1A). In contrast, HIF-1α was detected only in a few cells in the sublining and deep layer of human RA synovium (Figure 1A). However, neither HIF-1α nor HIF-2α was detected in human osteoarthritis (*n* = 10), psoriatic arthritis (*n* = 2), or gouty arthritis (*n* = 2) synovium (Figure S1B). These results indicate RA-specific differential up-regulation of HIF-1α and HIF-2α in synovial tissues.

We extended these results using the collagen-induced arthritis (CIA) model of RA in DBA/1J mice. This is a commonly used experimental model of inflammatory joint arthritis caused by a T-cell–dependent, antibody-mediated autoimmune response directed against cartilage type II collagen [16]. Compared with nonimmunized (NI) control joints, joints in CIA mice exhibited destruction typical of RA (Figure S1C–E). HIF-2α was highly up-regulated in the region lining the CIA synovium (Figure 1C), where it was co-localized with the RA-synovium markers, IL6, MMP3, and MMP13 (Figure 1B). Unlike HIF-2α expression, HIF-1α was rarely detected in the intimal lining, but was detected in cells of the sublining and deep layer (Figure 1C). Similar to human RA joint tissues, HIF-2α was also detected in pannus and damaged cartilage (Figure S1F). Quantitation of relative HIF expression levels further confirmed the marked up-regulation of HIF-2α compared with HIF-1α in human RA and mouse CIA synovia (Figure 1D). HIF-2α–positive cells were much more abundant in synovial lining cells (fibroblast-like and macrophage-like synoviocytes) compared with sublining macrophages and endothelial cells in blood vessels of RA synovium (Figure 1D).

### Overexpression of HIF-2α, But Not HIF-1α, in Joint Tissues Causes Experimental RA

**Figure 2:**
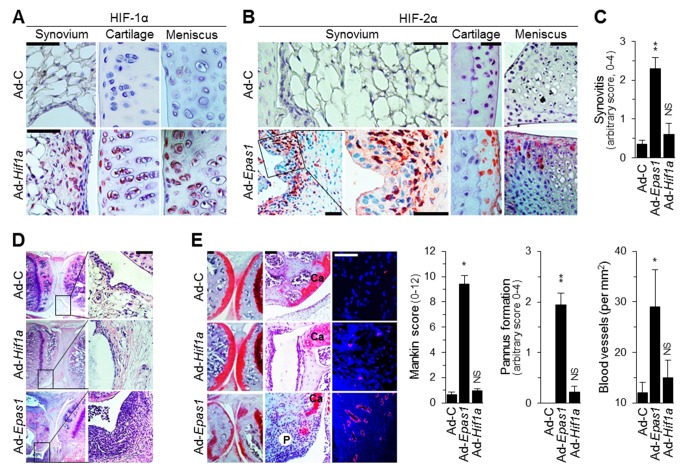
Overexpression of HIF-2α, but not HIF-1α, in joint tissues causes an RA-like phenotype in mice. DBA/1J mice were IA-injected with 1×10^9^ PFU of empty virus (Ad-C), Ad-*Epas1*, or Ad-*Hif1a*. After 3 wk, mice were sacrificed for further analysis. (A and B) Representative images of HIF-1α (A) and HIF-2α (B) immunostaining in knee joint tissues. (C and D) Scoring of synovial inflammation (*n* = 20) (C) and representative images of H&E staining (D). (E) Safranin-O staining and scoring of cartilage destruction (*n* = 20), safranin-O/hematoxylin staining and quantitation of pannus formation (*n* = 15), and CD31 staining and quantitation of blood vessels (*n* = 15) in Ad-injected knee joints. Ca, cartilage; P, pannus. Values are means ± SEM (*p<0.001, **p<0.0002). NS, not significant. Scale bars, 50 µm.

The expression patterns of HIF-1α and HIF-2α in RA synovium suggested differential roles of HIF isoforms. To explore the possible *in vivo* functions of HIFs, we overexpressed HIF-1α or HIF-2α in the knee joint tissues of DBA/1J mice via intra-articular (IA) injection of Ad-*Hif1a* or Ad-*Epas1* adenoviruses (1×10^9^ plaque-forming units [PFUs]), respectively. Immunostaining of joint tissue sections 3 wk after IA injection revealed that the respective adenoviruses caused marked overexpression of HIF-1α and HIF-2α in the synovium, cartilage, and meniscus of joint tissues (Figure 2A and B). HIF-2α expression in joint tissues caused typical RA-like phenotypic manifestations, including synovial hyperplasia and severe synovitis, determined by hematoxylin and eosin (H&E) staining and scoring of inflammation (Figure 2C and D); marked cartilage destruction, determined by safranin-O staining and scored by Mankin's method (Figure 2E); pannus formation and invasion into calcified cartilage and bone, determined by hematoxylin/safranin-O staining and scoring (Figure 2E); and angiogenesis in the synovium, determined by immunostaining for CD31 and counting blood vessels in synovia of knee and ankle joints (Figure 2E). Overexpressed HIF-2α in the synovium of Ad-*Epas1*–injected mice was co-localized with the RA-synovium marker IL6, as determined by double-immunofluorescence microscopy (Figure S1G). In contrast to HIF-2α, HIF-1α overexpression did not cause any changes in joint architecture, including hallmarks of RA such as synovitis, pannus formation, angiogenesis, and cartilage destruction (Figure 2C–E). Collectively, these results indicate that ectopic expression of HIF-2α, but not HIF-1α, causes typical RA-like joint destruction in mice, suggesting distinct functions of HIF-1α and HIF-2α in RA pathogenesis.

### HIF-2α Deficiency in Mice Inhibits Experimental RA

**Figure 3:**
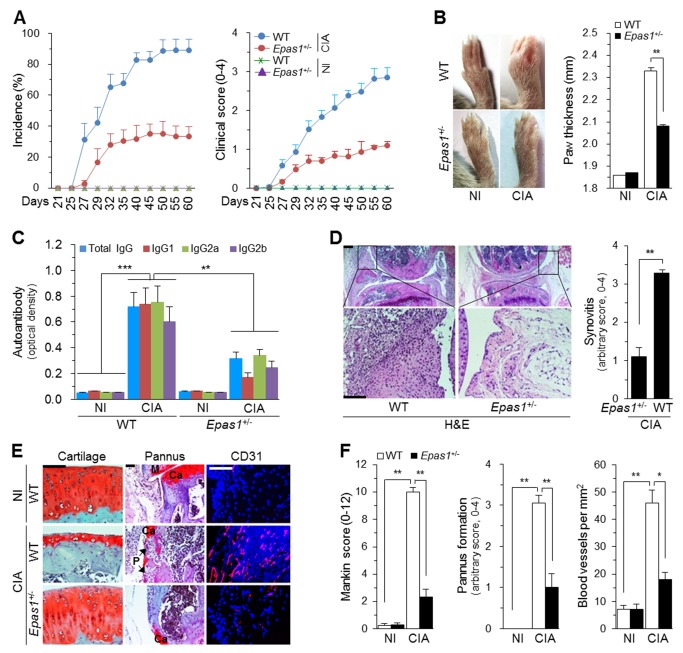
CIA is inhibited in *Epas1* ^+/−^ DBA/1J mice. (A) Incidence and severity of CIA in WT and *Epas1* ^+/−^ DBA/1J mice without (NI) and with CIA (*n* = 20 mice per group). (B) Typical paw images on day 60 after the first immunization and ankle thickness measured with a digital thickness caliper (*n* = 20 mice per group). (C) Type II collagen-specific autoantibody production under NI and CIA conditions in the sera of WT and *Epas1* ^+/−^ DBA/1J mice (*n* = 12). (D) H&E staining and scoring of synovial inflammation (*n* = 10). (E) Representative images of safranin-O staining of articular cartilage, safranin-O/hematoxylin staining of the pannus, and immunofluorescence microscopy of CD31 in knee joints of WT and *Epas1* ^+/−^ DBA/1J mice without (NI) and with CIA. (F) Quantification of results in (E). Mankin score (*n* = 12), pannus formation (*n* = 10), and number of blood vessels in the synovium (*n* = 10). Ca, cartilage; P, pannus; M, meniscus. Values are means ± SEM (*p<0.01, **p<0.001, ***p<0.0005). Scale bar, 50 µm.

We confirmed the role of HIF-2α using HIF-2α–knockout mice or local deletion of HIF-2α in joint tissues. We first examined HIF-2α functions using mice with reduced expression of the *Epas1* gene encoding HIF-2α. Because homozygous deletion of *Epas1* (*Epas1* ^−/−^) is embryonic lethal [17], we used heterozygous *Epas1* ^+/−^ mice. We have previously shown that deletion of one allele of *Epas1* is sufficient to inhibit OA cartilage destruction [18]. Whereas *Epas1* ^+/−^ DBA/1J mice showed reduced expression levels of HIF-2α mRNA, HIF-1α mRNA levels were not altered in these mice (unpublished data). Compared with wild-type (WT) littermates, *Epas1* ^+/−^ DBA/1J mice showed a significantly reduced incidence (89.4%±7.1% versus 33.2%±6.5%, *p* = 0.0004) and severity (2.85%±0.26% versus 1.10%±0.10%, *p* = 0.004) of CIA on day 60 after the first injection of type II collagen (Figure 3A). *Epas1* ^+/−^ DBA/1J mice under CIA conditions also showed a significant reduction in all the examined hallmarks of RA. These include paw swelling and increased ankle thickness (Figure 3B), elevated serum levels of autoantibodies against type II collagen (Figure 3C), synovitis (Figure 3D), cartilage destruction (Figure 3E and F), pannus formation and invasion (Figure 3E and F), and angiogenesis in inflamed synovium (Figure 3E and F).

**Figure 4:**
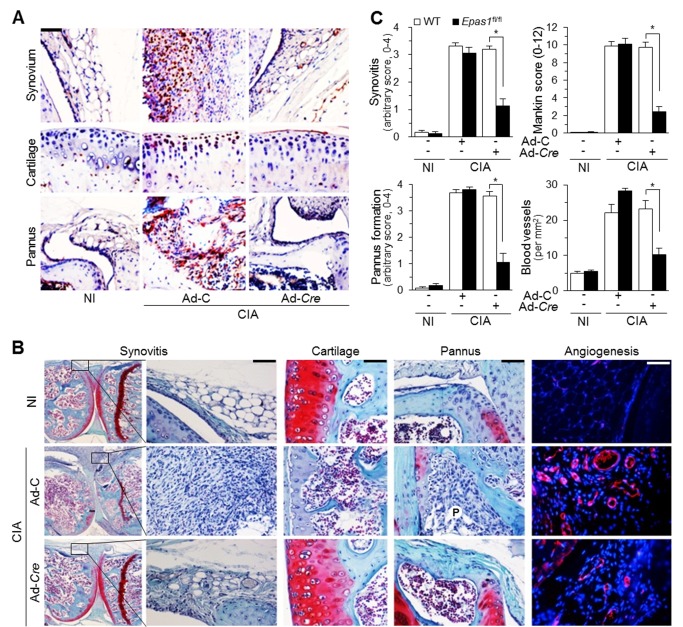
Local deletion of *Epas1* in joint tissues inhibits CIA. *Epas1* ^fl/fl^ mice were IA-injected with Ad-C or Ad-*Cre* (1×10^9^ PFU), immunized with type II collagen (CIA) or NI, and maintained for 3 wk. (A) HIF-2α in joint tissues was detected by immunostaining (*n* = 10). (B) Synovitis, cartilage destruction, pannus formation, and angiogenesis were detected by H&E staining, safranin-O staining, safranin-O/hematoxylin staining, and CD31 immunostaining, respectively. Representative images were obtained from more than 10 independent experiments. P, pannus. (C) Quantification of synovitis, pannus formation, blood vessels in the synovium, and Mankin score (*n*>10). Values are means ± SEM (*p<0.0005). Scale bar, 50 µm.

We further validated HIF-2α functions in CIA by locally deleting *Epas1* in joint tissues via IA injection of Ad-*Cre* (1×10^9^ PFU) in *Epas1* ^fl/fl^ mice. Immunostaining of joint sections revealed that Ad-*Cre* injection effectively reduced the elevated levels of HIF-2α induced by CIA in joint tissues, including synovium, cartilage, and pannus (Figure 4A). Moreover, local deletion of *Epas1* in joint tissues by Ad-*Cre* injection significantly inhibited RA pathogenesis by blocking synovitis and synovial hyperplasia, pannus formation and invasion into calcified cartilage and bone, angiogenesis in inflamed synovium, and cartilage destruction (Figure 4B and C). These results collectively indicate that *Epas1* knockdown (*Epas1* ^+/−^) or local deletion (Ad-*Cre*) inhibits experimental RA in mice.

### HIF-2α Modulates Immune Responses Without Affecting Immune System Development

**Figure 5:**
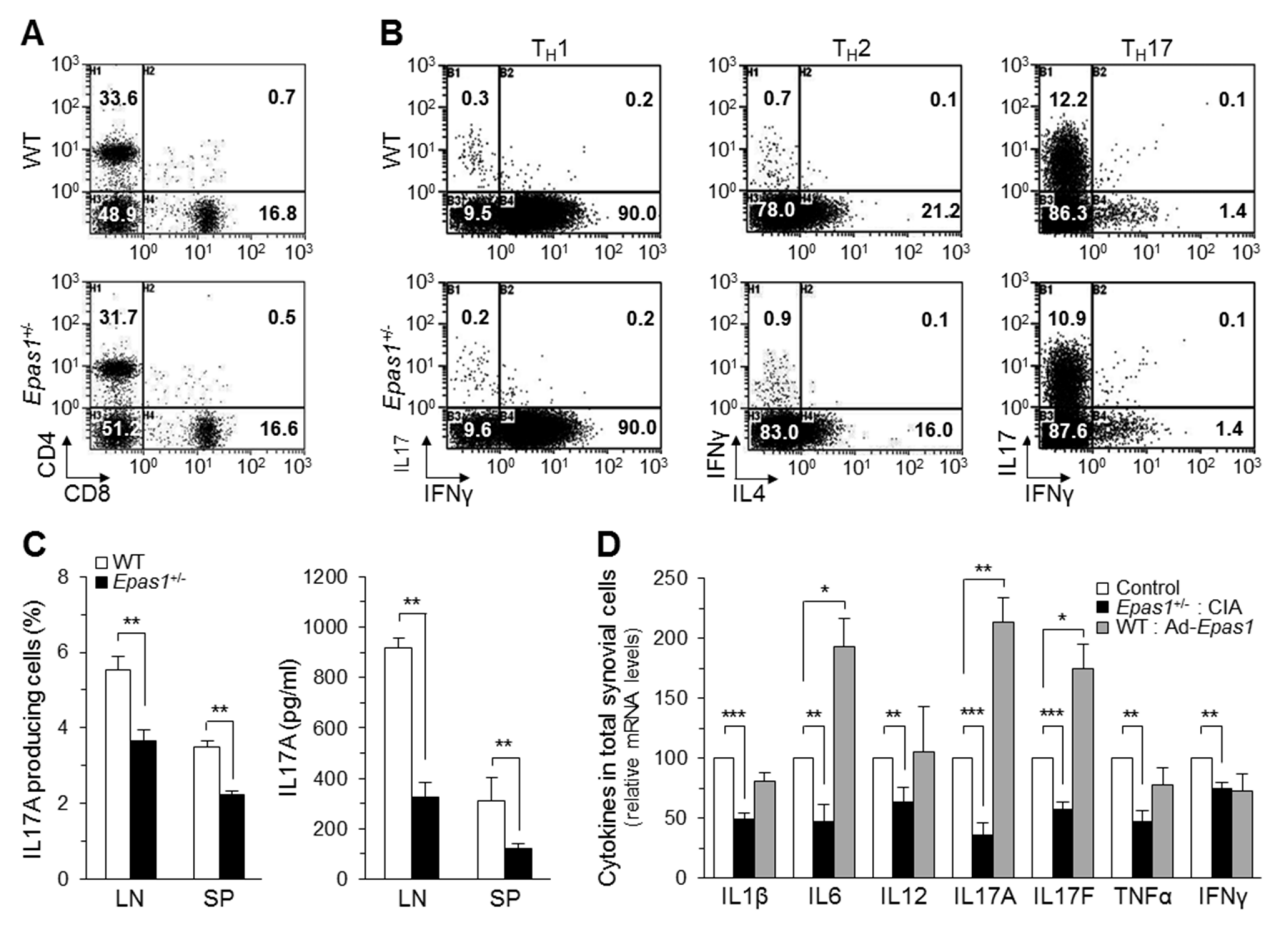
Normal immune system development and effector function of CD4^+^ T cells in *Epas1* ^+/−^ mice. (A) Representative flow cytometric analysis of CD4^+^ and CD8^+^ T-cell populations in the lymph nodes of WT and *Epas1* ^+/−^ DBA/1J mice. (B) Populations of T_H_1, T_H_2, and T_H_17 cells differentiated from uncommitted CD4^+^ T cells of WT and *Epas1* ^+/−^ DBA/1J mice. (C) IL17A-producing cells identified by flow cytometry (left), and levels of secreted IL17A determined by ELISA (right), from lymphocytes (LN) and splenocytes (SP) of WT and *Epas1* ^+/−^ DBA/1J mice (*n* = 8 mice per group) under CIA conditions. (D) mRNA levels of the indicated cytokines in total knee synovial cells isolated from *Epas1* ^+/−^ DBA/1J mice under CIA conditions or in Ad-*Epas1*–injected mice (*n* = 10). The NI condition and Ad-C injection were used as controls. Values are means ± SEM (*p<0.01, **p<0.005, ***p<0.0005).

Next, we investigated the inhibitory mechanisms of RA pathogenesis in *Epas1* ^+/−^ DBA/1J mice by examining immune responses. *Epas1* ^+/−^ mice showed normal populations of CD4^+^ and CD8^+^ T cells in lymph nodes, as determined by flow cytometry (Figure 5A). Flow cytometry also revealed no differences in immune cell populations between WT and *Epas1* ^+/−^ DBA/1J mice, including CD4^+^ and CD8^+^ T cells in spleen and thymus; Foxp3-expressing regulatory T cells (T_reg_) in lymph node, spleen, and thymus; naïve (CD44^low^CD62L^high^) and memory (CD44^high^CD62L^low^) CD4^+^ T cells in lymph node and spleen; and B220^+^ B cells and CD11c^+^ dendritic cells in lymph node and spleen (Figure S2A–D). Proliferation of CD4^+^ T cells and B220^+^ B cells isolated from lymph nodes and spleens was similar between WT and *Epas1* ^+/−^ DBA/1J mice (Figure S2E and F). Additionally, CD4^+^ T cells isolated from lymph nodes and spleens of *Epas1* ^+/−^ mice showed a normal capacity to differentiate into T_H_1, T_H_2, and T_H_17 cells, as determined by the detection of specific cytokines (Figure 5B and Figure S2G and H).

Although immune system development was not affected in *Epas1* ^+/−^ mice, HIF-2α knockdown in these mice modulated immune responses under CIA conditions. The population of IL17A–producing cells in lymph nodes and spleens as well as the levels of secreted IL17A, which plays a key role in T_H_17 cell differentiation and autoimmune responses, were significantly down-regulated under CIA conditions in *Epas1* ^+/−^ mice (Figure 5C). We further validated the effects of *Epas1* knockdown on pathogenic cytokine expression in synovial cells using a total mixed-cell population isolated from synovial tissues of WT and *Epas1* ^+/−^ mice. mRNA levels of the pathogenic cytokines IL1β, IL6, IL12, IL17A, IL17F, TNFα, and interferon (IFN)-γ under CIA conditions were significantly down-regulated in the total synovial cell population isolated from *Epas1* ^+/−^ mice compared with WT littermates (Figure 5D). Conversely, IA injection of Ad-*Epas1* (1×10^9^ PFU) significantly increased mRNA levels of IL6, IL17A, and IL17F in the total synovial cell population compared with those in Ad-C–injected mice (Figure 5D). Collectively, our results indicate that *Epas1* knockdown in *Epas1* ^+/−^ DBA/1J mice does not alter the development pattern of the immune system, but does significantly reduce the production of pathogenic cytokines under CIA conditions.

### HIF-2α Is Overexpressed in FLS of RA Synovium

**Figure 6:**
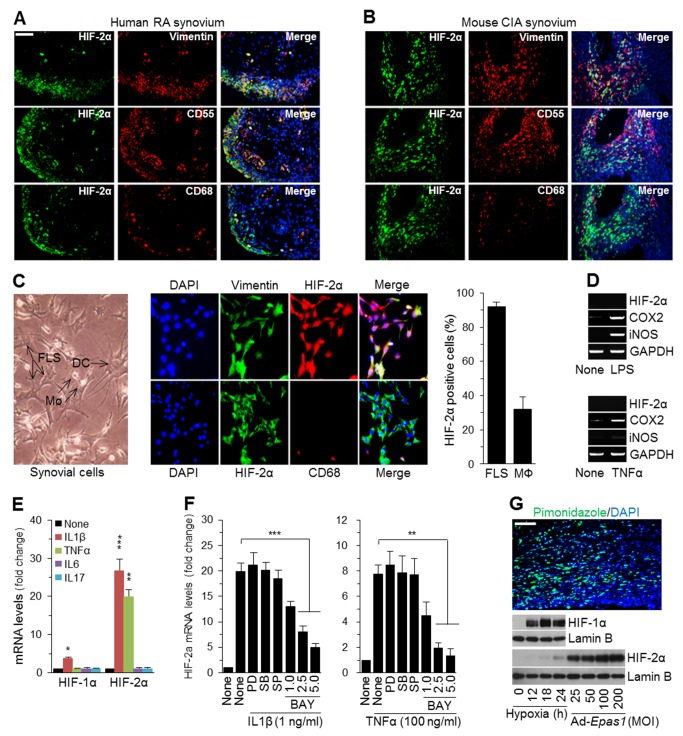
HIF-2α is up-regulated by pro-inflammatory cytokines in FLS of RA synovium. (A and B) Typical immunofluorescence microscopy images of DAPI, HIF-2α, and FLS markers (vimentin and CD55) or the macrophage marker CD68 in human RA synovium (A) and mouse CIA synovium (B). (C, Left) Primary culture of total synovial cells isolated from DBA/1J mice. DC, dendritic cells; MΦ, macrophages. (Center) Typical immunofluorescence microscopy images of HIF-2α, DAPI, and vimentin or CD68. (Right) The percentage of FLS and macrophages positive for HIF-2α staining was determined from six microscopic fields (*n* = 4). (D) Raw264.7 cells were treated with LPS (50 ng/ml) or TNFα (50 ng/ml) for 24 h. mRNA levels were detected by RT-PCR analysis (*n* = 6). (E) Primary cultured FLS were treated with IL1β (1 ng/ml), IL6 (100 ng/ml), IL17 (10 ng/ml), or TNFα (100 ng/ml) for 24 h. mRNA levels of HIF-1α and HIF-2α were quantified by qRT-PCR (*n* = 6). (F) FLS were treated with PD98059 (PD; 20 µM) to inhibit ERK, SB203580 (SB; 20 µM) to inhibit p38 MAP kinase, SP600125 (SP; 20 µM) to inhibit JNK, or the indicated concentration (µM) of BAY 11-7085 (BAY) to inhibit NF-κB. The cells were exposed to IL1β or TNFα for 24 h, and HIF-2α mRNA levels were quantified (*n* = 6). (G) Mouse CIA synovium was stained for the hypoxia marker pimonidazole (upper). Primary cultured FLS were maintained under hypoxic conditions or were infected with Ad-*Epas1* at an MOI of 800 for 24 h. HIF-1α and HIF-2α proteins were detected by Western blotting (lower). Values are presented as means ± SEM (*p<0.01, **p<0.005, ***p<0.001). Scale bar, 50 µm.

HIF-2α is up-regulated mainly in the intimal lining of RA synovium, which primarily consists of FLS and macrophage-like synoviocytes [4]. We therefore examined which cell types overexpress HIF-2α in inflamed RA synovium. Double-immunofluorescence microscopy of human RA (Figure 6A) and mouse CIA (Figure 6B) synovia revealed co-localization of HIF-2α with FLS markers (vimentin or CD55), whereas only a subset of CD68-positive macrophages expressed HIF-2α. We further examined HIF-2α expression in primary cultures of the total synovial cell population isolated from CIA mice; these cells consist of FLS, macrophages, and dendritic cells, among others (Figure 6C). Most FLS (∼92%) were positive for HIF-2α staining, whereas only ∼32% of macrophages were positive for HIF-2α staining (Figure 6C). To elucidate the role of HIF-2α expression in macrophages, we stimulated Raw264.7 cells (a murine macrophage cell line) with TNFα or lipopolysaccharide (LPS). Both stimuli caused up-regulation of the inflammatory mediators COX2 (cyclooxygenase 2) and iNOS (inducible nitric oxide synthase), without affecting HIF-2α expression (Figure 6D). These results collectively suggest that HIF-2α is mainly up-regulated in FLS of RA synovium, where it may play a major role in RA pathogenesis.

Next, we investigated the mechanisms regulating HIF-2α expression using primary cultures of mouse FLS. The pro-inflammatory cytokines IL1β and TNFα induced up-regulation of HIF-2α in FLS, whereas IL6 and IL17 did not affect HIF-2α expression (Figure 6E). A pharmacological analysis using inhibitors of nuclear factor–kappaB (NF-κB) and mitogen-activated protein (MAP) kinase subtypes indicated that IL1β- and TNFα-induced HIF-2α expression in FLS is mediated by the NF-κB pathway, but not by the MAP kinase pathway (Figure 6F). Because hypoxia is a prominent feature of the inflamed RA synovium [5]–[7], we additionally examined the role of hypoxia in HIF-2α expression in FLS. Mouse CIA synovium was hypoxic, as determined by pimonidazole staining (Figure 6G). However, unlike HIF-1α protein levels, which were markedly elevated in FLS under hypoxic conditions, HIF-2α protein showed only minimal accumulation under the same conditions; however, Ad-*Epas1* infection under normoxic conditions caused marked expression of HIF-2α protein (Figure 6G). Collectively, these findings suggest that pro-inflammatory cytokines, rather than hypoxia, are the leading cause of HIF-2α expression in FLS under CIA conditions.

### HIF-2α Regulates RA-Associated FLS Functions

**Figure 7:**
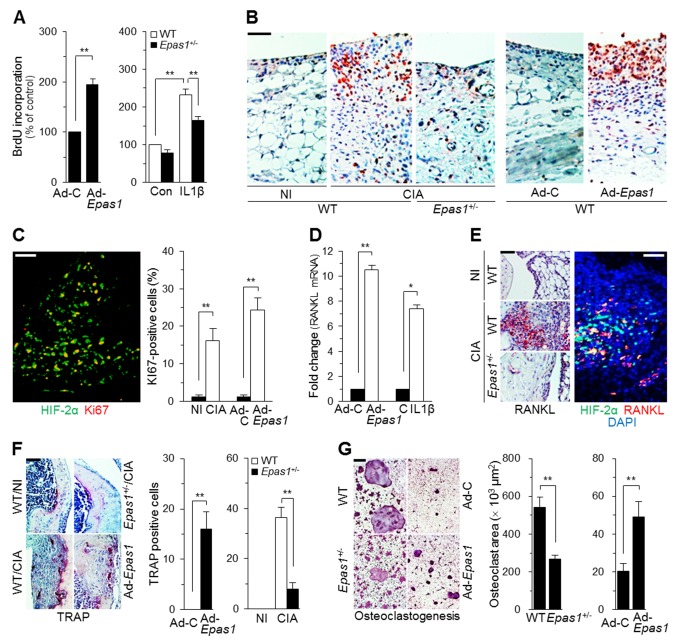
HIF-2α regulates FLS proliferation, RANKL expression in FLS, osteoclastogenesis, and pannus formation. (A) BrdU-incorporation assays in FLS infected with Ad-C or Ad-*Epas1* (MOI 800) (left), and FLS from WT or *Epas1* ^+/−^ mice treated with 1 ng/ml of IL1β (right) (*n* = 6). (B) Ki67 staining in synovial sections from WT and *Epas1* ^+/−^ mice without (NI) or with CIA, or from WT mice injected with Ad-C or Ad-*Epas1* (MOI 800) (*n* = 8). (C) Double-immunofluorescence staining for HIF-2α and Ki67 in mouse CIA synovium. Ki67-positive cells among HIF-2α–overexpressing cells were counted (*n* = 8). (D) RANKL mRNA levels were quantified in FLS infected with Ad-C or Ad-*Epas1* (MOI 800) or treated with 1 ng/ml of IL1β (*n* = 10). (E) Representative images of RANKL immunostaining in the knee synovium of WT or *Epas1* ^+/−^ mice without (NI) or with CIA (Left). Typical immunofluorescence microscopy image of triple-stained CIA synovium (Right). (F) TRAP staining and counting of TRAP-positive multinucleated cells (*n* = 10) in the pannus of the bone–cartilage interface in WT and *Epas1* ^+/−^ mice without (NI) or with CIA, or following injection with 1×10^9^ PFU of Ad-C or Ad-*Epas1*. (G) TRAP staining during *in vitro* osteoclastogenesis of precursor cells isolated from WT and *Epas1* ^+/−^ mice or WT precursor cells infected with Ad-C or Ad-*Epas1* (800 MOI) (Left). Osteoclastogenesis was quantified by measuring the osteoclast area (*n* = 10) (Right). Values are means ± SEM (*p<0.01, **p<0.001). Scale bar, 50 µm.

FLS play a crucial role in RA pathogenesis by producing various regulatory factors [4]. We therefore explored whether up-regulated HIF-2α in FLS modulates FLS functions and thereby RA pathogenesis. Because increased survival and/or proliferation of FLS contribute to synovial hyperplasia [4], we first examined HIF-2α regulation of apoptosis and proliferation in these cells. Ad-*Epas1*–mediated HIF-2α overexpression in primary cultured FLS did not cause apoptosis or modulate apoptosis induced by an anti-Fas antibody (unpublished data) known to cause FLS apoptosis [4]. However, HIF-2α overexpression significantly increased proliferation of FLS, and IL1β-induced proliferation was inhibited in *Epas1* ^+/−^ FLS (Figure 7A). Moreover, staining for the cell proliferation marker Ki67 revealed the presence of proliferating cells in the intimal lining of both CIA and Ad-*Epas1*–infected synovia; notably, this staining was markedly reduced in *Epas1* ^+/−^ DBA/1J mice (Figure 7B). Double immunostaining for HIF-2α and Ki67 indicated that 16% and 24% of HIF-2α–positive cells were proliferative in inflamed synovia caused by CIA and Ad-*Epas1* injection, respectively (Figure 7C).

Pannus formation and invasion into adjacent cartilage and bone are important regulatory steps in cartilage and bone erosion, which is mediated by the actions of osteoclasts [1],[3],[4]. Osteoclastogenesis is regulated by RANKL, which is produced by FLS and T cells, and requires physical contact of precursor cells with RANKL-expressing FLS or T cells in RA synovium [3],[19]. We therefore examined a possible role for HIF-2α in FLS regulation of RANKL expression, osteoclastogenesis, and pannus formation. HIF-2α overexpression or IL1β treatment of FLS caused significant up-regulation of RANKL mRNA levels (Figure 7D). Additionally, immunostaining indicated markedly increased levels of RANKL protein in CIA synovium of WT mice, an effect that was reduced in *Epas1* ^+/−^ mice (Figure 7E). HIF-2α and RANKL were co-localized in CIA synovium, as determined by double immunostaining (Figure 7E). Consistent with this, TRAP staining revealed an increase in the number of multinucleated osteoclasts in the pannus of the bone–cartilage interface of CIA and Ad-*Epas1*–infected joints of WT mice; this too was also significantly reduced in *Epas1* ^+/−^ mice (Figure 7F). The role of HIF-2α in osteoclastogenesis was further determined using *Epas1* ^+/−^ precursor cells, with and without HIF-2α overexpression. Osteoclastogenesis of *Epas1* ^+/−^ precursor cells was significantly reduced compared with that of WT cells (Figure 7G). Moreover, overexpression of HIF-2α in precursor cells by Ad-*Epas1* infection enhanced osteoclastogenesis (Figure 7G). These results collectively support the idea that HIF-2α–mediated production of RANKL in FLS and osteoclastogenesis of precursor cells contribute to cartilage and bone erosion during HIF-2α–induced RA pathogenesis.

### HIF-2α Up-Regulates Catabolic Factor Expression in FLS

**Figure 8:**
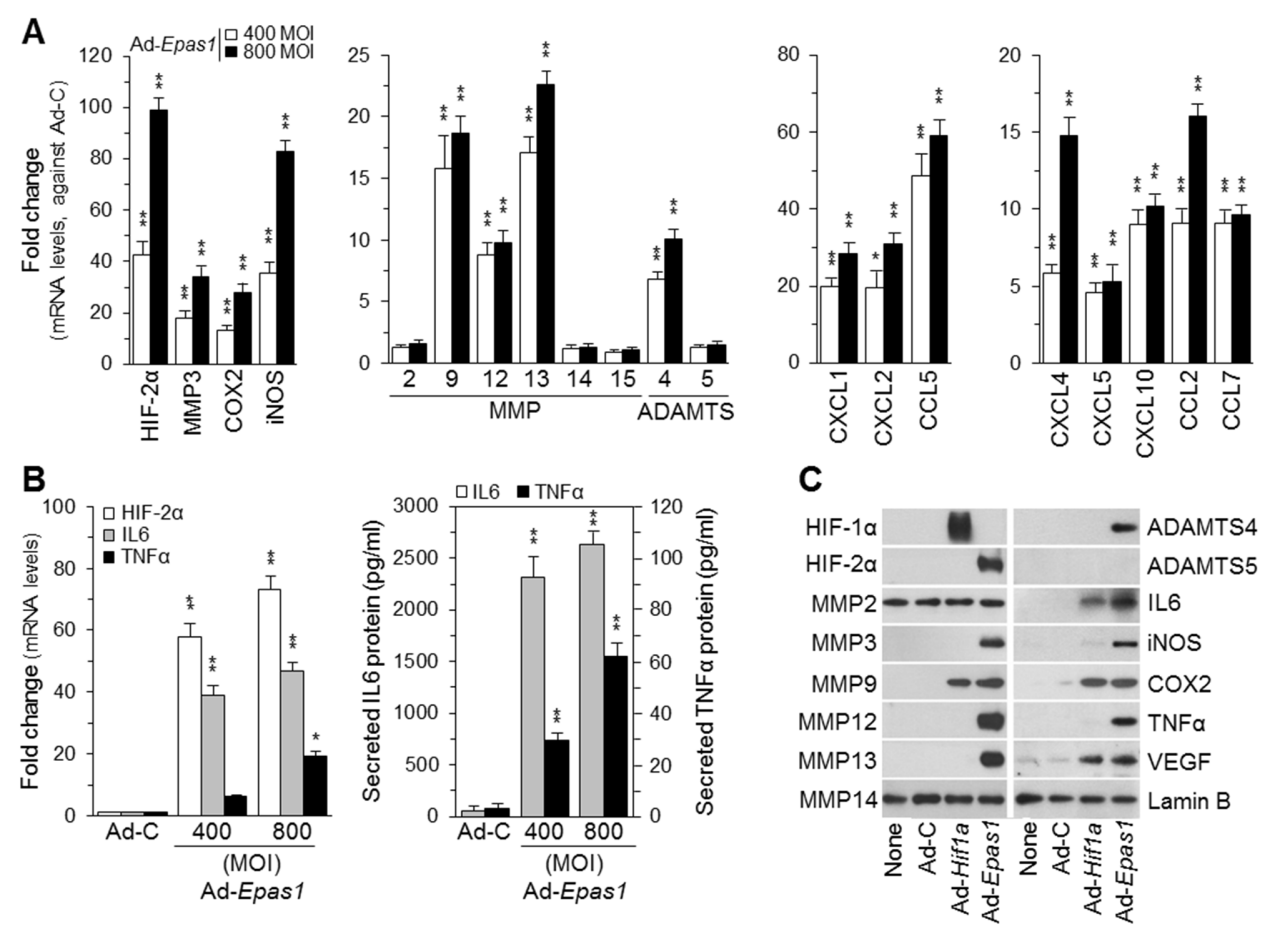
HIF-2α up-regulates the expression of cytokines, chemokines, and catabolic enzymes in FLS. (A) qRT-PCR analysis (*n*≥8) of catabolic enzymes and chemokines in FLS infected with Ad-C (800 MOI) or with Ad-*Epas1* at the indicated MOI for 24 h. (B) qRT-PCR analysis (*n*≥8) of mRNA levels of HIF-2α, IL6, and TNFα (Left), and ELISA of secreted IL6 and TNFα proteins (Right) in FLS infected with Ad-C (800 MOI) or with Ad-*Epas1* at the indicated MOI for 24 h. (C) FLS were infected with Ad-C, Ad-*Epsa1*, or Ad-*Hif1a* (800 MOI) for 24 h. The indicated proteins were detected by Western blotting (*n* = 5). Values are means ± SEM (*p<0.01, **p<0.001).

FLS regulate RA pathogenesis by producing various cytokines, chemokines, and matrix-degrading enzymes involved in inflammation, chemotaxis, cartilage destruction, and bone erosion [4]. This led us to explore a possible role for HIF-2α in the expression of these regulatory factors. Ad-*Epas1*–infected FLS exhibited significantly increased mRNA levels of matrix-degrading enzymes (MMP3, MMP9, MMP12, MMP13, and ADAMTS4), chemokines (CCL2, CCL5, CCL7, CXCL1, CXCL2, CXCL4, CXCL5, and CXCL10), and inflammatory mediators (COX2 and iNOS) (Figure 8A). Among the cytokines examined (IL1β, IL6, IL11, IL12, IL17, IL21, LIF, and TNFα), both mRNA and protein levels of IL6 and TNFα were increased in response to HIF-2α overexpression (Figure 8B and C). Moreover, IL1β-induced up-regulation of catabolic factors (matrix-degrading enzymes, cytokines, and chemokines) was abolished by the knockdown of *Epas1* with two independent small interfering RNAs (siRNAs) (Figure S3). In contrast to the effects of HIF-2α, overexpression of HIF-1α in FLS caused up-regulation of MMP9, IL6, COX2, and VEGF, but not that of other factors regulated by HIF-2α (Figure 8C).

### HIF-2α–Induced Up-Regulation of IL6 in FLS Regulates T_H_17 Cell Differentiation

**Figure 9:**
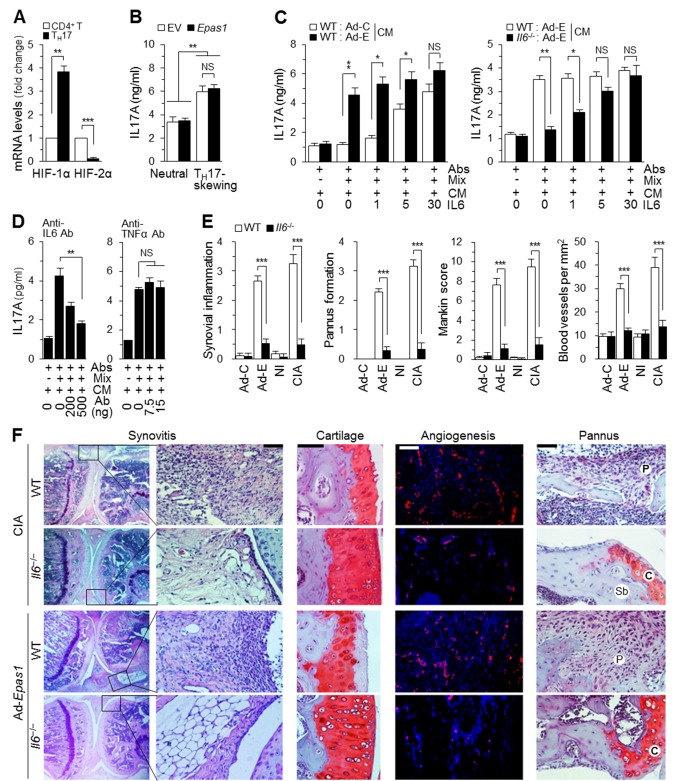
HIF-2α–derived IL6 in FLS regulates T_H_17 cell differentiation and RA pathogenesis. (A) HIF-1α and HIF-2α mRNA levels in CD4^+^ T cells and differentiated T_H_17 cells, determined by qRT-PCR (*n* = 5). (B) Mouse CD4^+^ T cells were transfected with empty vector (EV) or *Epas1*-expressing vector, and cultured under neutral or T_H_17-skewing conditions. T_H_17 cell differentiation was evaluated by monitoring IL17A production (*n* = 4). (C) Precursor CD4^+^ T cells were left untreated or were treated with antibodies against CD3 and CD28 (Abs), Mix (i.e., TGFβ, IL2, and antibodies against IL4, IFNγ, and IL12), the indicated amount of IL6, or CM from WT FLS infected with Ad-C or Ad-*Epas1* (Ad-E) at an MOI of 800 (left), or WT and *Il6^−/−^* FLS infected with Ad-*Epas1* (Ad-E; 800 MOI) (Right). T_H_17 cell differentiation was evaluated by monitoring IL17A production (*n* = 8). (D) T_H_17 cell differentiation in the presence of Abs, Mix, or CM from WT FLS infected with Ad-*Epas1* (Ad-E) and/or the indicated amounts of neutralizing antibody against IL6 or TNFα was evaluated by monitoring IL17A (*n* = 6). (E and F) Synovial inflammation, pannus formation, angiogenesis, and cartilage destruction were detected by joint tissue staining and quantified in WT and *Il6^−/−^* DBA/1J mice injected with Ad-C or Ad-*Epas1* (1×10^9^ PFU) or without (NI) or with CIA. Values are means ± SEM (*p<0.01, **p<0.001, ***p<0.0005). Scale bar, 50 µm.

T_H_17 cells are crucial effectors of RA pathogenesis [2],[3], and HIF-1α has been previously shown to regulate T_H_17 cell differentiation [13],[14]. For instance, enhanced HIF-1α expression in T_H_17 cells positively regulates T_H_17 differentiation by up-regulating RORγt, an isoform of RAR-related orphan receptor gamma [13]. We therefore examined possible functions of HIF-2α in T_H_17 cell differentiation, and thereby RA pathogenesis. We first examined mRNA levels of HIF-1α and HIF-2α during T_H_17 cell differentiation. Compared with precursor CD4^+^ T cells, differentiated T_H_17 cells exhibited significant down-regulation of HIF-2α expression and significant up-regulation of HIF-1α expression (Figure 9A). Unlike the case with HIF-1α, which enhances T_H_17 cell differentiation [13], overexpression of HIF-2α in precursor CD^+^ T cells did not affect T_H_17 cell differentiation (Figure 9B), suggesting that HIF-2α in CD4^+^ T cells does not directly modulate T_H_17 cell differentiation.

It is well established that IL6 plays a key role in T_H_17 cell differentiation [2],[3]. Consistent with this, *in vitro* T_H_17 cell differentiation was dependent on the addition of exogenous IL6 protein (Figure 9C). Given the marked overexpression of IL6 in FLS induced by HIF-2α, we explored possible functions of HIF-2α–regulated, FLS-derived IL6 in T_H_17 cell differentiation by treating CD4^+^ precursor T cells with conditioned medium (CM) prepared from Ad-C (control)- or Ad-*Epas1*–infected FLS. T_H_17 cell differentiation was evaluated by monitoring IL17A production using an enzyme-linked immunosorbent assay (ELISA). As shown in Figure 9C, addition of CM from Ad-*Epas1*–infected FLS from WT mice induced T_H_17 cell differentiation, even in the absence of exogenous IL6 protein. The specific role of IL6 in CM was confirmed by preparing CM from FLS of *Il6* ^−/−^ mice or by adding IL6 neutralizing antibody to the CM. Compared with CM from WT FLS, CM of Ad-*Epas1*–infected FLS from *Il6* ^−/−^ mice did not affect *in vitro* T_H_17 cell differentiation (Figure 9C). Furthermore, addition of a neutralizing antibody against IL6, but not TNFα, blocked stimulation of T_H_17 cell differentiation by the CM of Ad-*Epas1*–infected FLS (Figure 9D). We additionally confirmed T_H_17 cell differentiation by monitoring mRNA levels of IL17A and IL17F using quantitative reverse transcription–polymerase chain reaction (qRT-PCR) analysis (Figure S4). Immunostaining of synovial sections also revealed the presence of IL17A-producing cells in mouse synovium infected with Ad-*Epas1* or under CIA conditions, whereas no positive immunostaining was observed in Ad-C–infected or NI synovium (Figure S5A–C). Indeed, IL17A-positive cells were located in close proximity to HIF-2α–positive cells in human RA and mouse CIA synovia, as determined by double immunostaining (Figure S5D).

### HIF-2α Does Not Cause a RA Phenotype in *Il6* ^−/−^ Mice

The above results suggest that FLS-derived IL6 plays an important role in HIF-2α regulation of experimental RA by regulating T_H_17 cell differentiation. To confirm this, we investigated IL6 functions in HIF-2α–induced experimental RA using *Il6* ^−/−^ mice. Consistent with the inhibition of CIA by *Il6* knockout [20],[21], we also observed significantly greater inhibition of synovitis, pannus formation and invasion, cartilage destruction, and angiogenesis in inflamed synovium under CIA conditions in *Il6* ^−/−^ DBA/1J mice compared with WT littermates (Figure 9E and F). More importantly, the development of RA-like phenotypic manifestations, including synovitis, pannus formation and invasion, cartilage destruction, and angiogenesis, induced in inflamed synovium by IA injection of Ad-*Epas1* was markedly diminished in *Il6* ^−/−^ DBA/IJ mice compared with WT mice (Figure 9E and F). Our results collectively suggest that FLS-derived IL6 plays an important role in T_H_17 cell differentiation and thereby contributes to HIF-2α regulation of experimental RA.

## Discussion

Our current findings provide two novel insights into the regulation of RA pathogenesis by HIF pathways: the catabolic role of HIF-2α in RA pathogenesis and the differential actions of HIF-1α and HIF-2α in this disease.

In the first case, we demonstrate an essential role for HIF-2α in the pathogenesis of RA. Despite circumstantial evidence for the hypoxic status of RA synovium [5]–[7] and increased expression of HIF-2α in the synovial lining of human RA patients [9], little is currently known about the role of HIF-2α in RA pathogenesis. The results of our loss-of-function studies utilizing *Epas1* knockdown in mice (*Epas1* ^+/−^) or local deletion in *Epas1* ^fl/fl^ mice by Ad-*Cre* injection strongly support our conclusion that HIF-2α is necessary for RA pathogenesis. This conclusion is reinforced by the marked up-regulation of HIF-2α observed in RA synovia of humans and mouse models of RA as well as the RA-like phenotype revealed in gain-of-function studies involving IA injection of Ad-*Epas1*. In RA joint tissues, HIF-2α is up-regulated in various tissues, including synovium, pannus, cartilage, meniscus, and TRAP-positive osteoclasts. IA injection of Ad-*Epas1* also caused up-regulation of HIF-2α in these tissues. Because numerous cell types in joint tissues contribute to the process of RA pathogenesis [1], up-regulated HIF-2α in any of these tissues could contribute to RA pathogenesis. However, because HIF-2α levels were most markedly increased in synovial cells, which are also the primary targets of adenovirus infection, we characterized HIF-2α functions in synovial tissue in the regulation of RA development. In RA synovial tissue, HIF-2α was up-regulated in most FLS in the synovium lining compartment, although some other cell types, such as macrophages, also exhibited HIF-2α up-regulation. Although we cannot rule out a contribution of these other cell types, we were able to demonstrate that HIF-2α regulates RA-associated FLS functions in experimental RA pathogenesis. These include proliferation; expression of cytokines, chemokines, and matrix-degrading enzymes; RANKL expression and osteoclastogenesis; IL6 production; and IL6-dependent T_H_17 cell differentiation. Among these, IL6-dependent T_H_17 cell differentiation is a crucial effector of RA pathogenesis. In this context, we demonstrated that IL6 present in CM prepared from FLS caused T_H_17 cell differentiation. Moreover, IL17A-positive cells were located in close proximity to HIF-2α–positive cells, suggesting that IL6 production mediated by HIF-2α in the inflamed RA synovium affects differentiation of neighboring T_H_17 cells. Additional support for this relationship is provided by our demonstration that global deletion of *Il6* abolished HIF-2α–induced RA pathogenesis. Although it remains possible that production of IL6 by cell types in synovial tissue besides FLS could also contribute to the regulation of T_H_17 cell differentiation, establishing this definitively would likely require a conditional FLS-specific *Il6*-knockout model, which, to our knowledge, has not yet been developed.

The second novel finding of this study is that HIF-1α and HIF-2α have distinct roles and act via different mechanisms in RA pathogenesis. HIF-1α is up-regulated in RA synovium [10]–[12], where it is associated with angiogenesis [5]–[7]. It has previously been demonstrated that HIF-1α regulates RA pathogenesis by directly modulating T_H_17 cell functions [13],[14]. In the current study, HIF-1α expression, in contrast to that of HIF-2α, was detected in a small number of cells in the sublining and deep layer of RA synovium in both humans and experimental mouse models of RA, a result consistent with other reports [10],[11]. We did not extensively explore the underlying mechanisms of this differential expression of HIF isoforms in the current study. However, HIF-1α and HIF-2α show different sensitivity to oxygen tension and display distinct, and sometimes opposing, cellular activities [8],[9]. Indeed, we found in this study that sensitivities to hypoxia and to pro-inflammatory cytokines differed between HIF-1α and HIF-2α in FLS. These differences may reflect the differential expression pattern of HIF-2α and HIF-2α in RA synovium. Nevertheless, ectopic expression of HIF-1α in joint tissues by IA injection of Ad-*Hif1a* did not cause an RA-like phenotype, suggesting that HIF-1α overexpression is not sufficient to induce RA pathogenesis. In striking contrast, HIF-2α overexpression was sufficient to activate RA pathogenesis and did so by regulating FLS functions. Collectively, our results suggest that HIF-2α regulates RA pathogenesis by acting globally to modulate the RA pathogenesis program, including angiogenesis and FLS functions, whereas HIF-1α contributes to RA pathogenesis by modulating the effector functions of myeloid and T cells. Moreover, the observation that HIF-2α deficiency, which does not affect HIF-1α expression, is sufficient to inhibit experimental RA underscores the specific roles played by HIF-2α.

RA and OA are the most common types of joint arthritis. We have previously shown that HIF-2α is a catabolic regulator of OA cartilage destruction [18],[22]–[24], demonstrating that HIF-2α causes OA pathogenesis by up-regulating catabolic enzymes such as MMP3 and MMP13 in chondrocytes, and further showing that chondrocyte-specific *Col2a1*-*Epas1* TG mice exhibit spontaneous cartilage destruction with no evidence of synovitis [18]. Although RA and OA phenotypes share certain features, such as cartilage destruction, their etiology and pathogenesis are completely different. RA and OA also differ with respect to outcomes, cell types associated with the pathogenesis, and therapeutic approaches. For instance, OA is a degenerative joint disease (“wear and tear” arthritis) that begins with the destruction of surface articular cartilage, subchondral bone sclerosis, and osteophyte formation in a single joint. In this type of arthritis, mechanical stresses, including joint instability and injury, and factors that predispose toward OA, such as aging, are important causes of pathogenesis [25],[26]. In contrast to OA, RA is a systemic autoimmune disorder, which manifests as chronic inflammation that results in destruction of cartilage and bone tissues [2]–[4]. The inflammatory process initially affects a single joint, but the disease usually progresses to affect nearly all joints [27]. Thus, our results indicate that, despite their different etiologies and pathogenesis, both RA and OA are regulated by HIF-2α via completely different mechanisms: HIF-2α regulates OA pathogenesis by up-regulating matrix-degrading catabolic enzymes in articular chondrocytes, whereas it appears to regulate RA pathogenesis by regulating angiogenesis, various functions of FLS, and IL6-dependent T_H_17 cell differentiation.

In summary, our current studies suggest that HIF-2α is an essential catabolic regulator of RA pathogenesis that acts by modulating various RA-associated FLS functions. Because the etiology of RA pathogenesis has not yet been entirely elucidated and effective treatment of RA remains a significant unmet medical need, HIF-2α may serve as an effective therapeutic target in RA treatment. In this context, an important question that remains to be evaluated is whether recently developed small-molecule inhibitors of HIF-2α [28],[29] inhibit RA pathogenesis *in vitro* and *in vivo*. Additionally, because HIF-1α and HIF-2α appear to regulate RA pathogenesis through different mechanisms, both HIF isoforms could be alternative therapeutic targets in the treatment of RA disease.

## Materials and Methods

### Ethics Statement

The use of human materials was approved by the Institutional Review Board of Chonnam National University Hospital and Wonkwang University Hospital, and written informed consent was obtained from all individuals before the operative procedure. Mice were housed in specific pathogen-free barrier facilities and were used in accordance with protocols approved by the Animal Care and Ethics Committees of the Gwangju Institute of Science and Technology.

### Human Arthritic Joint Tissues

Human RA, psoriatic arthritis, gouty arthritis, and OA joint tissues were collected from patients undergoing knee arthroplasty (Tables S1, S2, S3) and then embedded in paraffin. All RA patients had a median disease duration of ∼6 y, high disease activity (i.e., median DAS of 5.61), and received medications, including a variety of disease-modifying antirheumatic drugs (Table S1). Because joint tissues were obtained from patients undergoing knee arthroplasty, our samples represent relatively late-stage RA.

### Mice and Experimental Arthritis

Male DBA/1J, C57BL/6, *Epas1* ^+/−^, *Epas1* ^fl/fl^, and *Il6^−/−^* mice were used for experimental RA studies. The C57BL/6 strains of *Epas1* ^+/−^, *Epas1* ^fl/fl^, and *Il6* ^−/−^ mice were described previously [18],[22]. *Epas1* ^+/−^, *Epas1* ^fl/fl^, and *Il6* ^−/−^ (C57BL/6) mice were backcrossed against the DBA/1J strain for eight generations to generate *Epas1* ^+/−^ DBA/1J, *Epas1* ^fl/fl^ DBA/1J, and *Il6* ^−/−^ DBA/1J mice, respectively. CIA was produced in WT and *Epas1* ^+/−^ DBA/1J mice using a standard protocol [16]. Briefly, mice were intradermally injected at the base of the tail with incomplete Freund's adjuvant alone (control) or Freund's adjuvant containing 100 µg of collagen type II; a booster injection was given 21 d later. *Epas1* ^fl/fl^ DBA/1J mice were IA-injected with Ad-C or Ad-*Cre* (1×10^9^ PFU) on days 0, 3, and 6, followed by a booster injection with collagen type II. Mice were maintained for an additional 2 wk. The incidence and severity of RA were evaluated on the indicated days after the first immunization. Severity was evaluated using a clinical score (grade 0–4) of paw swelling based on the level of inflammation in each of the four paws [16]. Joint tissues from mice were fixed, decalcified with 0.5 M EDTA (pH 8.0), embedded in paraffin, and sectioned at 5-µm thickness. Synovitis was evaluated by H&E staining of joint sections, and synovial inflammation (grade 0–4) was scored as described by Tang et al. [30]. The pannus in joint tissues adjacent to cartilage and bone was visualized by H&E staining with or without safranin-O staining of cartilage, and pannus formation was scored (grade 0–4) as described by Tang et al. [30]. Cartilage destruction was examined by safranin-O staining and scored using Mankin's method, as previously described [18],.

### Immunohistochemistry and Immunofluorescence Microscopy

Human and mice joint tissues were sectioned at 5-µm thickness for immunohistochemical staining. Antigen retrieval was performed by incubating sections with 0.1% trypsin for 40 min at 37°C or with citrate buffer for 20 min at 95°C. The following primary antibodies were used for immunohistochemistry: rabbit anti–HIF-2α and rabbit anti-RANKL (Santa Cruz), rabbit anti-MMP3 and anti-MMP13 (Abcam), goat anti-IL6 (R&D Systems), rabbit anti-IL17A (Abcam), mouse anti-HIF-1α (Sigma), and rat anti-CD31 (Dianova). For double-immunofluorescence labeling of human and mouse joint tissues, the following primary antibodies were used: rabbit anti–HIF-2α (Novus Biologicals for human tissues and Santa Cruz for mouse tissues), rabbit anti-MMP3 (Abcam), mouse anti-MMP13 (Calbiochem, for human synovia), rabbit anti-MMP13 (Abcam, for mouse synovia), goat anti-IL6 (R&D Systems), mouse anti-vimentin (BD Biosciences), rabbit anti-CD55 (Santa Cruz), rabbit anti-CD68 (Abcam, for human synovia), rat anti-CD68 (Abcam, for mouse synovia), rabbit anti-TRAP (Santa Cruz), rabbit anti-VEGF (Santa Cruz), rabbit anti-IL17A (Santa Cruz), rabbit anti-Ki67 (Abcam), and rabbit anti-RANKL (Santa Cruz). Expression levels of HIF-2α in RA synovium were quantified using Image J software. The percentage of cells expressing HIF-2α was analyzed in synovial lining cells (fibroblast-like and macrophage-like synoviocytes), sublining macrophages, and endothelial cells [32]. Cell types were distinguished according to their characteristic morphology and confirmed by immunoreactivity with anti-vimentin (FLS), anti-CD68 (macrophages), and anti-CD31 (endothelial cells) antibodies. For immunofluorescence staining of total synovial cells or FLS cultured on coverslips, the following antibodies were used: rabbit anti–HIF-2α (Novus Biologicals), goat anti-IL6 (R&D Systems), rabbit anti-CD68, and mouse anti-vimentin (BD Biosciences).

### FLS Culture, CM Preparation, and Proliferation Assays

Total synovial cells were isolated from knee joint synovium of CIA mice. Synovial tissues were minced and digested in collagenase for 4 h at 37°C. The cells were plated on coverslips in RPMI-1640 medium and incubated for 4 d. FLS were isolated from NI and CIA joint tissues of WT and *Il6* ^−/−^ mice [33]. FLS between passage 4 and 8 were used for further analysis. Pure FLS (>90% CD90^+^/<1% CD14^+^) were identified by flow cytometry using antibodies against the fibroblast marker CD90 and the macrophage marker CD14 (Abcam). For the preparation of CM, FLS were infected with Ad-C or Ad-*Epas1* at a multiplicity of infection (MOI) of 800 for 2 h and incubated on 35-mm culture dishes containing 1 ml of RPMI-1640 medium. CM was used to treat CD4^+^ precursor T cells during differentiation into T_H_17 cells. FLS proliferation in culture was quantified by measuring BrdU incorporation during DNA synthesis. Proliferating cells in synovial sections were identified by detecting Ki67 using an antibody obtained from Novus Biologicals.

### T-Cell Differentiation and Proliferation

CD4^+^ T cells from WT and *Epas1* ^+/−^ mice were purified from lymph nodes and spleens. T_H_ cell differentiation was induced by plating cells (2×10^6^ cells/ml) on culture dishes coated with anti-CD3 antibody (1 µg/ml) in the presence of soluble anti-CD28 antibody (2 µg/ml) under the following T_H_ cell-skewing conditions: T_H_1 cells, IL12 (10 ng/ml) and anti-IL4 antibody (10 µg/ml); T_H_2 cells, IL4 (20 ng/ml) and 10 µg/ml of antibodies against IFNγ and IL12; T_H_17 cells, tumor growth factor (TGF)-β (3 ng/ml), IL6 (30 ng/ml), and 10 µg/ml of antibodies against IL4, IFNγ, and IL12. IL2 (100 U/ml) was added after 24 h, and cells were cultured for 6 d. Antibodies and cytokines were purchased from BD Biosciences or PeproTech. The cells were stimulated with PMA (50 ng/ml), ionomycin (1 µM), and brefeldin A (1 mg/ml; eBioscience). T_H_ cell differentiation was evaluated by flow cytometry after staining for intracellular cytokines and by detecting cytokines by ELISA and qRT-PCR. Where indicated, activated CD4^+^ T cells were transfected by electroporation with empty vector or vector carrying *Epas1*. The cells were cultured under neutralizing conditions (10 µg/ml of antibodies against IL4 and IFNγ) or T_H_17 cell-skewing conditions for 4 d. The effects of HIF-2α overexpression on skewed CD4^+^ T cells were evaluated by detecting IL17A production by ELISA. For cell proliferation assays, CD4^+^ T and B220^+^ B cells were isolated from lymph nodes and spleens of WT and *Epas1* ^+/−^ DBA/1J mice. T-cell proliferation was induced by stimulating cells with anti-CD3 antibody (10 µg/ml), and B-cell proliferation was induced by stimulating cells with LPS (10 µg/ml), LPS plus IL4 (5 ng/ml), or antibodies against IgM (20 µg/ml; Jackson ImmunoResearch) and CD40 (10 µg/ml; BioLegend). Proliferation was assessed by measuring [^3^H]thymidine (0.5 µCi/well) incorporation during the last 18 h of a 72-h culture period.

### Hypoxia in FLS and Joint Tissues

For detection of the hypoxic status of mouse CIA synovium, mice immunized with type II collagen were intraperitoneally injected with hypoxyprobe-1 (pimonidazole HCl; Hypoxyprobe Inc.) at a dosage of 60 mg/kg body weight and sacrificed 6 h after injection. Paraffin-embedded joint tissues were sectioned at 5-µm thickness, and pimonidazole was detected by immunofluorescence microscopy, according to the manufacturer's instructions. For hypoxic culture of mouse FLS, cells were exposed to hypoxia for 12, 18, or 24 h in a GasPak anaerobic chamber (BBL GasPak Pouch; Becton Dickinson) at 37°C, as described previously [18]. The proportion of oxygen in each chamber was ≤1%.

### Flow Cytometric Analysis

Leukocytes were prepared from lymph nodes draining the inflamed joint, spleen, and thymus of NI and CIA mice. Synovial cells were harvested by digesting synovial tissues with collagenase. The cells were incubated with primary antibodies for 15 min at 4°C. Antibodies against CD4, CD8, CD44, Foxp3, B220, and CD11c were purchased from eBioscience; the anti-CD62L antibody was from BD Pharmingen. Nonspecific staining was ascertained using isotype-matched control antibodies. T_H_ cells were fixed in fixation/permeabilization buffer for 30 min; resuspended in 100 µl of permeabilization buffer; incubated for 30 min at 4°C with Alexa 488- or phycoerythrin (PE)-conjugated anti-IFNγ (eBioscience), fluorescein isothiocyanate (FITC)-conjugated anti-IL4, PE-conjugated anti-IL17A or isotype control antibodies (eBioscience); and analyzed by flow cytometry using EPICS XL and EXPO32 software (Beckman Coulter).

### ELISA of Cytokines and Autoantibody Production

Representative cytokines involved in RA pathogenesis (IFNγ, TNFα, IL4, and IL17A) and produced by T_H_ subsets were detected using ELISA kits (eBioscience), according to the manufacturer's protocol. IL6 and TNFα secreted into serum-free culture media by FLS infected with Ad-C or Ad-*Epas1* were quantified by ELISA. Collagen type II–specific antibodies were measured by ELISA. Sera from NI and CIA mice were added into 96-well plates coated with type II collagen (5 µg/ml), incubated overnight at 4°C, washed, and incubated for 1 h with alkaline phosphatase-labeled monoclonal antibodies against mouse IgG1, IgG2a, or IgG2b (Immunology Consultants Lab). Wells were developed using *p-*nitrophenyl phosphate as a substrate, and the resulting color reaction was quantified using an ELISA plate reader.

### Bone Marrow Culture, Osteoclastogenesis, and TRAP Staining

Bone marrow cell culture, osteoclastogenesis, and TRAP staining were performed as described previously [34]. Briefly, bone-marrow–derived macrophages were isolated from WT or *Epas1* ^+/−^ mice, seeded in 48-well plates (4×10^4^ cells/well), and cultured for 4 d (Ad-C and Ad-*Epas1* infection in WT cells) or 5 d (WT and *Epas1* ^+/−^ precursor cells) with M-CSF (macrophage colony stimulating factor; 30 ng/ml) and RANKL (100 ng/ml) to induce osteoclastogenesis. The surface area of TRAP-stained multinuclear osteoclasts containing three or more nuclei was measured using an Osteomeasure system (Osteometrics). TRAP activity was also determined in paraffin sections of joint tissues from NI and CIA mice or mice IA-injected with 1×10^9^ PFU of Ad-C or Ad-*Epas1*. The numbers of TRAP-positive osteoclasts and their precursor cells were counted in a blinded fashion in all regions of pannus-formed bone–cartilage interface and synovium for each knee joint.

### siRNA, RT-PCR, qRT-PCR, and Western Blotting

PCR primers and experimental conditions are summarized in Table S4. Two different siRNA sequences that silenced *Epas1* effectively were used in this study (Table S5). Nonsilencing, scrambled siRNA was used as a negative control. Cells were transfected for 6 h with siRNA using Lipofectamine 2000 (Invitrogen), and then infected with Ad-*Epas1* or treated with IL1β. In qRT-PCR, the relative levels of target mRNA were normalized to those of glyceraldehyde 3-phosphate dehydrogenase. The following antibodies were used for Western blotting: rabbit anti-MMP2, -3, -9, -12, -13, and -14 (Epitomics); rabbit anti-ADAMTS4 (Abcam); goat anti-IL6 (R&D Systems); rabbit anti-ADAMTS5 (Thermo Scientific); rabbit anti-iNOS, rabbit anti-VEGF, goat anti-RANKL, and goat anti-TNFα (Santa Cruz); and mouse anti-COX2 (Cayman Chemical).

### Statistical Analysis

The nonparametric Mann–Whitney *U* test was used for the analysis of data based on an ordinal grading system, such as synovitis, pannus, and Mankin scores. For results obtained in qRT-PCR assays, ELISAs, and analyses of blood vessel numbers, joint thickness, TRAP-positive cells, BrdU incorporation, thymidine incorporation, and apoptotic cell numbers, data were first tested for conformation to a normal distribution using the Shapiro–Wilk test and then were analyzed by Student's *t* test (pair-wise comparisons) or analysis of variance (ANOVA) with post hoc tests (multicomparison), as appropriate. Significance was accepted at the 0.05 level of probability (*p*<0.05).

## Supporting Information

Figure S1 Up-regulation of HIF-2α in RA joint tissues of humans and mice. (A) Human RA bone sections were immunostained for HIF-2α and TRAP, and counterstained with DAPI. Damaged and undamaged parts of human RA cartilage were stained for HIF-2α (*n* = 4). (B) HIF-2α immunostaining in osteoarthritis (OA; *n* = 10), gouty arthritis (GA; *n* = 2), and psoriatic arthritis (PsA; *n* = 2) synovial sections. (C) Clinical score in DBA/1J mice immunized with type II collagen (CIA) or NI (*n* = 20 mice per group). (D) Sections of knee and ankle joints of NI and CIA mice obtained 6 wk after the first immunization. (Left) H&E staining; (Right) synovial inflammation scores (*n* = 15). (E) Joint sections were collected 6 wk after the first injection and stained with safranin-O (*n* = 15) to detect cartilage destruction. (F) Typical images of HIF-2α immunostaining in joint sections of DBA/1J mice 6 wk after the first immunization (*n* = 15). (G) Typical image of triple-stained (HIF-2α, IL6, and DAPI) synovial sections from mice IA-injected with Ad-*Epas1* (1×10^9^ PFU). Values are means ± SEM. C, cartilage; S, synovium; M, meniscus; P, pannus; Sb, subchondral bone. Scale bar, 50 µm.
(TIF)

Figure S2 *Epas1* knockdown in mice (*Epas1* ^+/−^) does not affect immune system development. (A–D) Leukocytes were isolated from the indicated tissues of naïve WT and *Epas1* ^+/−^ DBA/1J mice. Cells were stained for markers of various immune cell types, and immune cell populations were determined by flow cytometry and quantified (*n*>10 mice). CD4^+^ helper T cells and CD8^+^ cytotoxic T cells (A); Foxp3-expressing regulatory T cells (T_reg_) (B); naïve (CD44^low^CD62L^high^) and memory (CD44^high^CD62L^low^) CD4^+^ T cells (C); B220^+^ B cells and CD11c^+^ dendritic cells (D). (E and F) CD4^+^ T cells (E) and B220^+^ B cells (F) were isolated from lymph nodes and spleens from WT and *Epas1* ^+/−^ DBA/1J mice (*n*>8 mice). Proliferation of cells cultured for 3 d in the absence or presence of the appropriate T- or B-cell stimulants was assessed by [^3^H]thymidine incorporation assays. Results are expressed as counts per minute (CPM). (G and H) CD4^+^ T cells were purified from the lymph nodes and spleens of WT and *Epas1* ^+/−^ DBA/1J mice. T_H_ cell differentiation was induced under T_H_1-, T_H_2-, or T_H_17-skewing conditions. Recombinant IL2 (100 U/ml) was added after 24 h, and cells were cultured in complete medium for 6 d. Cells were restimulated with PMA, ionomycin and brefeldin A, or left untreated, and then stained for intracellular cytokines. The indicated cytokines were detected by ELISA in untreated cells (G) and restimulated cells (H) (*n* = 6). Values are presented as means ± SEM.
(TIF)

Figure S3 Knockdown of *Epas1* by siRNA inhibits IL1β-induced catabolic factor expression in primary culture FLS. (A and B) FLS were left untreated (None) or were treated with 100 nM control siRNA (C-siRNA) or the indicated amounts of two different *Epas1*-specific siRNAs—siRNA-1 (A) or siRNA-2 (B)—and then were exposed to IL1β for an additional 24 h. mRNA levels of the indicated catabolic factors were quantified by qRT-PCR (*n* = 10). Values are means ± SEM (*p<0.05, **p<0.01, ***p<0.001 compared with C-siRNA treatment).
(TIF)

Figure S4 IL6 produced by FLS regulates T_H_17 cell differentiation. T_H_17 cell differentiation was evaluated by detecting mRNA levels of IL17A (A) and IL17F (B) (*n* = 6). Precursor CD4^+^ T cells were left untreated or were treated with antibodies against CD3 and CD28 (Abs), Mix (i.e., TGFβ, IL2, and antibodies against IL4, IFNγ, and IL12), the indicated amount of IL6, or CM from WT FLS infected with Ad-C or Ad-*Epas1* (800 MOI), or WT and *Il6^−/−^* FLS infected with Ad-*Epas1* (800 MOI). T_H_17 cell differentiation was evaluated by monitoring IL17A expression (left panels). T_H_17 cell differentiation in the presence of CM from WT FLS infected with Ad-*Epas1* and/or the indicated amounts of neutralizing antibodies against IL6 or TNFα was evaluated by monitoring IL17A (right panels). Values are means ± SEM (*p<0.05, **p<0.005, ***p<0.0005).
(TIF)

Figure S5 *Epas1* knockdown in mice (*Epas1* ^+/−^) inhibits IL17 expression. (A) CIA was produced in WT and *Epas1* ^+/−^ DBA/1J mice, and IL17 protein was detected in NI and CIA synovial sections after 6 wk by immunohistochemistry. (B) The knee joints of WT and *Epas1* ^+/−^ DBA/1J mice were injected with Ad-C or Ad-*Epas1* (1×10^9^ PFU). After 3 wk, synovial sections were immunostained for IL17 and counterstained with hematoxylin. (C) IgG was used as a negative immunostaining control. Representative images are shown (*n* = 6). (D) Typical images of triple-stained (HIF-2α, IL17A, and DAPI) human RA synovium and mouse CIA synovium (*n* = 6). Scale bar, 50 µm.
(TIF)

Table S1 Clinical characteristics of RA patients with synovial biopsy.
(DOCX)

Table S2 Clinical characteristics of two patients with gouty arthritis.
(DOCX)

Table S3 Clinical characteristics of two patients with psoriatic arthritis.
(DOCX)

Table S4 PCR primers and conditions.
(DOCX)

Table S5 siRNA sequences.
(DOCX)

## Abbreviations

CIA: collagen-induced arthritis
CM: conditioned medium
FLS: fibroblast-like synoviocytes
HIF: hypoxia-inducible factor
H&E: hematoxylin and eosin
HUVEC: human umbilical vein endothelial cell
IA: intra-articular
IL: interleukin
MMP: matrix metalloproteinase
MOI: multiplicity of infection
OA: osteoarthritis
PFUs: plaque-forming units
qRT-PCR: reverse transcription–polymerase chain reaction
RA: rheumatoid arthritis
RANKL: receptor activator of nuclear factor–κB ligand
TNF: tumor necrosis factor; TRAP, tartrate-resistant acid phosphatase
VEGF: vascular endothelial growth factor
