# Supplementary figures and images for: Hypoxia-Inducible Factor-2α Is an Essential Catabolic Regulator of Inflammatory Rheumatoid Arthritis
Source: PLoS Biol. 2014 Jun 10;12(6):e1001881. doi: 10.1371/journal.pbio.1001881 (PMC4051611; doi:10.1371/journal.pbio.1001881)

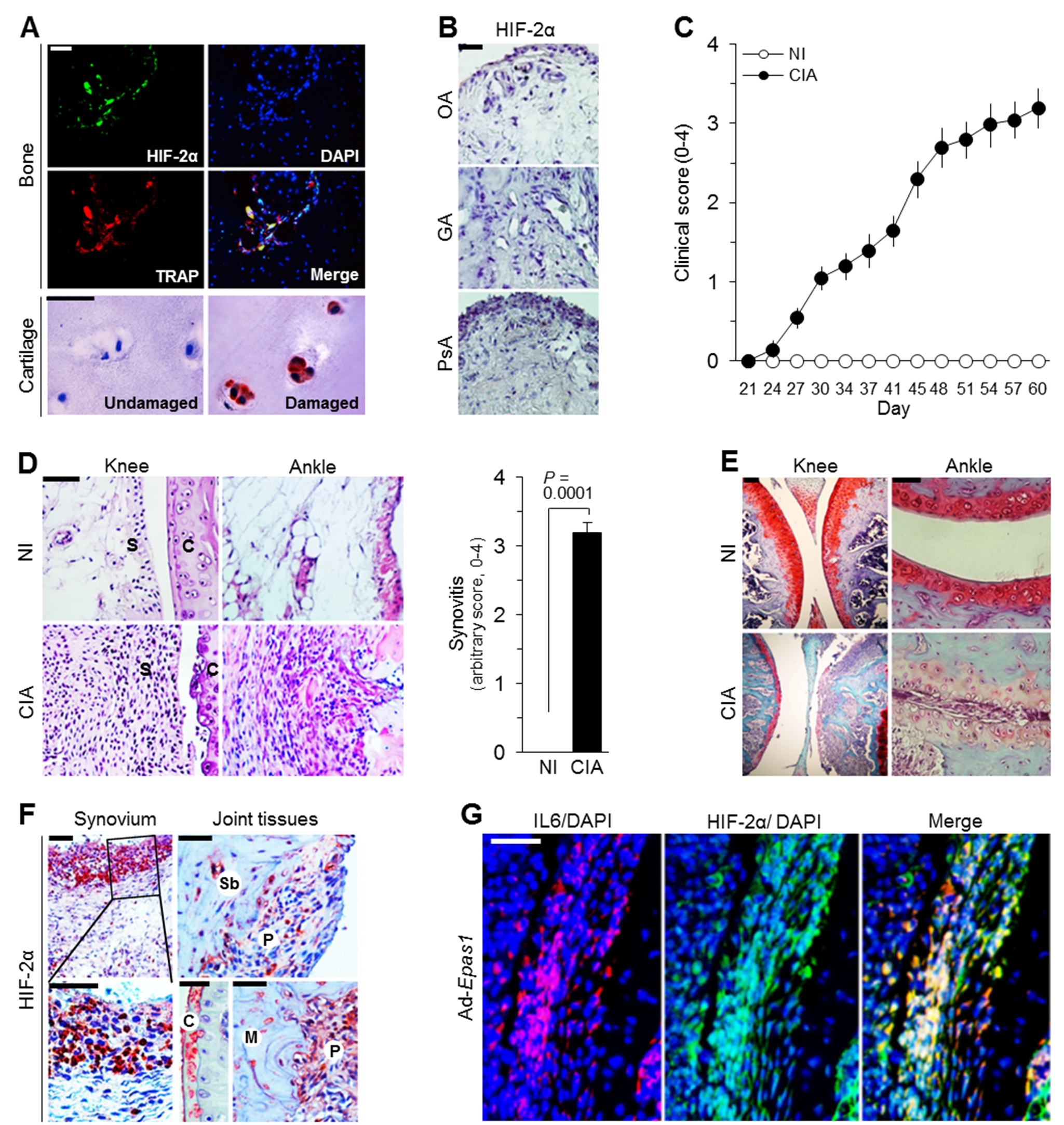

Supplement: Figure S1 — Up-regulation of HIF-2α in RA joint tissues of humans and mice. (A) Human RA bone sections were immunostained for HIF-2α and TRAP, and counterstained with DAPI. Damaged and undamaged parts of human RA cartilage were stained for HIF-2α (n = 4). (B) HIF-2α immunostaining in osteoarthritis (OA; n = 10), gouty arthritis (GA; n = 2), and psoriatic arthritis (PsA; n = 2) synovial sections. (C) Clinical score in DBA/1J mice immunized with type II collagen (CIA) or NI (n = 20 mice per group). (D) Sections of knee and ankle joints of NI and CIA mice obtained 6 wk after the first immunization. (Left) H&E staining; (Right) synovial inflammation scores (n = 15). (E) Joint sections were collected 6 wk after the first injection and stained with safranin-O (n = 15) to detect cartilage destruction. (F) Typical images of HIF-2α immunostaining in joint sections of DBA/1J mice 6 wk after the first immunization (n = 15). (G) Typical image of triple-stained (HIF-2α, IL6, and DAPI) synovial sections from mice IA-injected with Ad-Epas1 (1×10^9^ PFU). Values are means ± SEM. C, cartilage; S, synovium; M, meniscus; P, pannus; Sb, subchondral bone. Scale bar, 50 µm. (TIF) [file pbio.1001881.s001.tif]

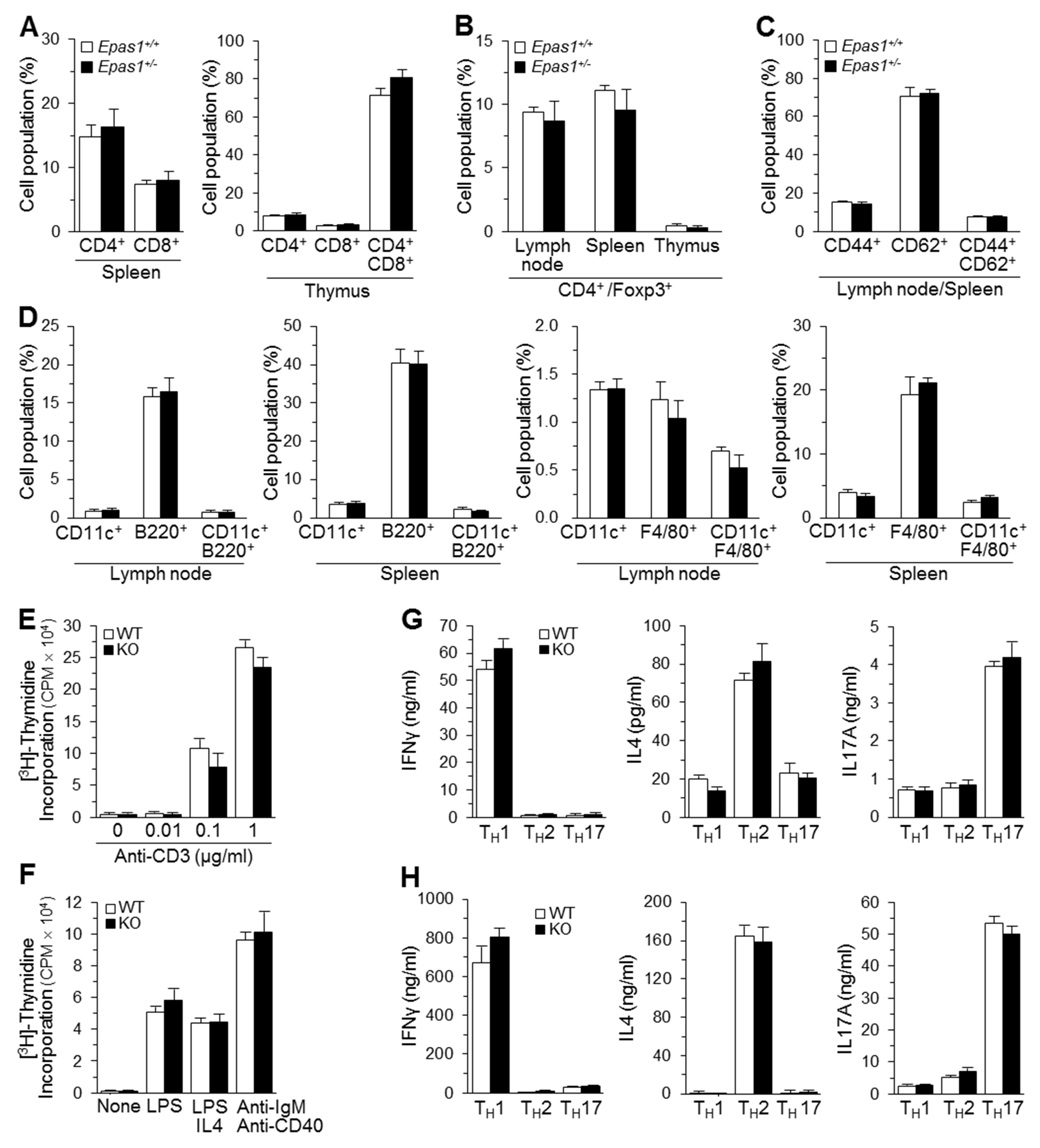

Supplement: Figure S2 — Epas1 knockdown in mice (Epas1 +/−) does not affect immune system development. (A–D) Leukocytes were isolated from the indicated tissues of naïve WT and Epas1 +/− DBA/1J mice. Cells were stained for markers of various immune cell types, and immune cell populations were determined by flow cytometry and quantified (n>10 mice). CD4+ helper T cells and CD8+ cytotoxic T cells (A); Foxp3-expressing regulatory T cells (Treg) (B); naïve (CD44lowCD62Lhigh) and memory (CD44highCD62Llow) CD4+ T cells (C); B220+ B cells and CD11c+ dendritic cells (D). (E and F) CD4+ T cells (E) and B220+ B cells (F) were isolated from lymph nodes and spleens from WT and Epas1 +/− DBA/1J mice (n>8 mice). Proliferation of cells cultured for 3 d in the absence or presence of the appropriate T- or B-cell stimulants was assessed by [3H]thymidine incorporation assays. Results are expressed as counts per minute (CPM). (G and H) CD4+ T cells were purified from the lymph nodes and spleens of WT and Epas1 +/− DBA/1J mice. TH cell differentiation was induced under TH1-, TH2-, or TH17-skewing conditions. Recombinant IL2 (100 U/ml) was added after 24 h, and cells were cultured in complete medium for 6 d. Cells were restimulated with PMA, ionomycin and brefeldin A, or left untreated, and then stained for intracellular cytokines. The indicated cytokines were detected by ELISA in untreated cells (G) and restimulated cells (H) (n = 6). Values are presented as means ± SEM. (TIF) [file pbio.1001881.s002.tif]

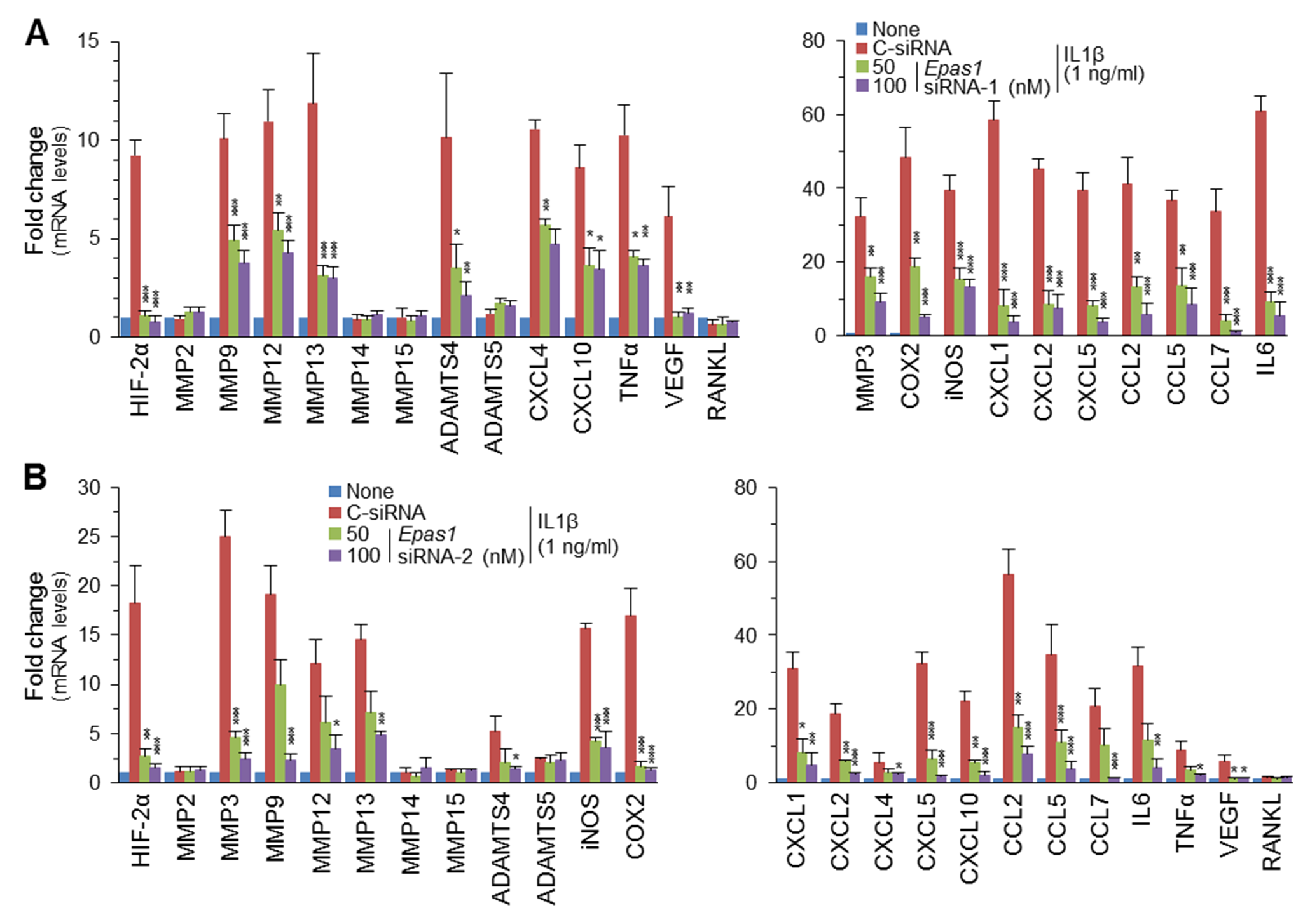

Supplement: Figure S3 — Knockdown of Epas1 by siRNA inhibits IL1β-induced catabolic factor expression in primary culture FLS. (A and B) FLS were left untreated (None) or were treated with 100 nM control siRNA (C-siRNA) or the indicated amounts of two different Epas1-specific siRNAs—siRNA-1 (A) or siRNA-2 (B)—and then were exposed to IL1β for an additional 24 h. mRNA levels of the indicated catabolic factors were quantified by qRT-PCR (n = 10). Values are means ± SEM (*p<0.05, **p<0.01, ***p<0.001 compared with C-siRNA treatment). (TIF) [file pbio.1001881.s003.tif]

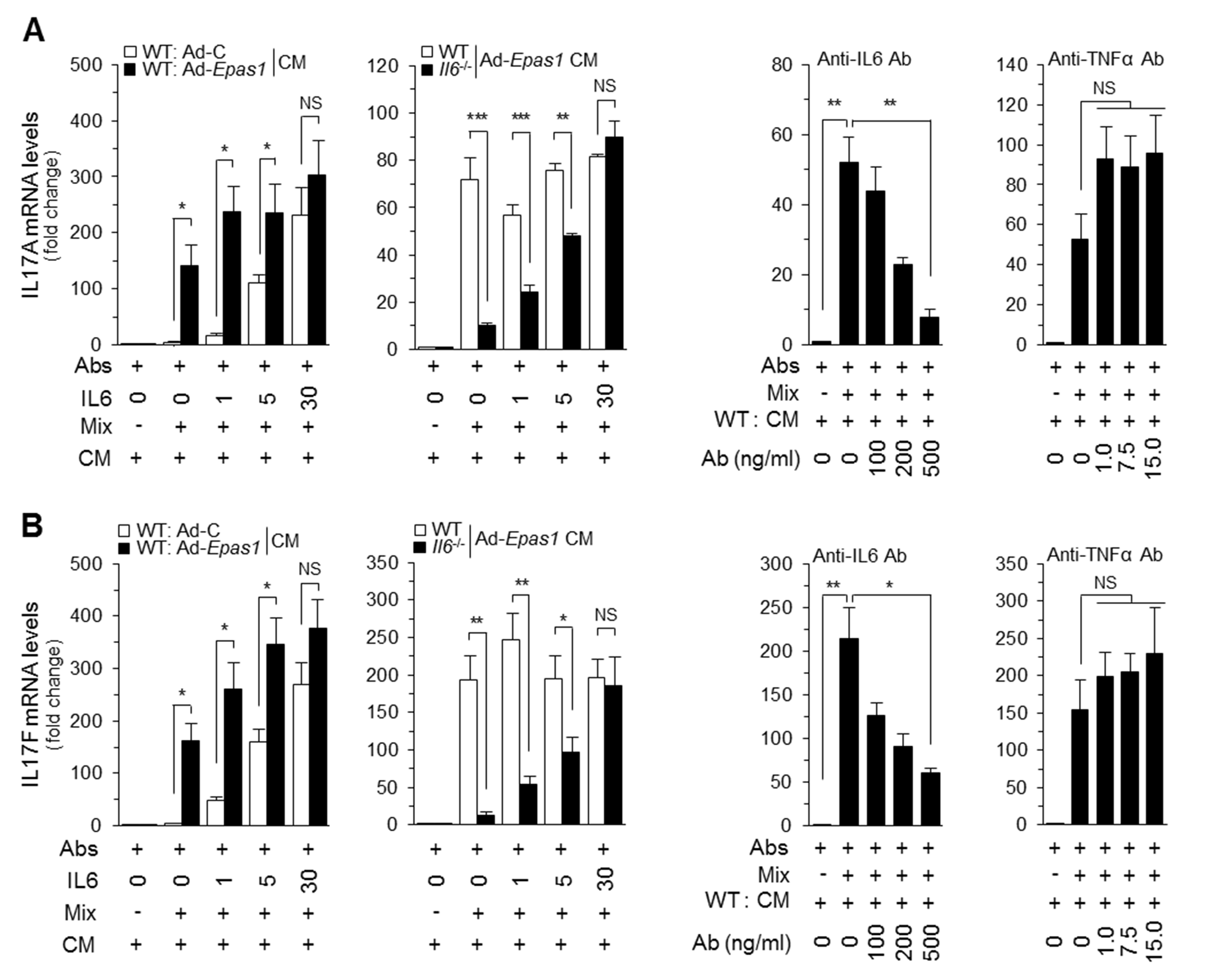

Supplement: Figure S4 — IL6 produced by FLS regulates TH17 cell differentiation. TH17 cell differentiation was evaluated by detecting mRNA levels of IL17A (A) and IL17F (B) (n = 6). Precursor CD4+ T cells were left untreated or were treated with antibodies against CD3 and CD28 (Abs), Mix (i.e., TGFβ, IL2, and antibodies against IL4, IFNγ, and IL12), the indicated amount of IL6, or CM from WT FLS infected with Ad-C or Ad-Epas1 (800 MOI), or WT and Il6−/− FLS infected with Ad-Epas1 (800 MOI). TH17 cell differentiation was evaluated by monitoring IL17A expression (left panels). TH17 cell differentiation in the presence of CM from WT FLS infected with Ad-Epas1 and/or the indicated amounts of neutralizing antibodies against IL6 or TNFα was evaluated by monitoring IL17A (right panels). Values are means ± SEM (*p<0.05, **p<0.005, ***p<0.0005). (TIF) [file pbio.1001881.s004.tif]

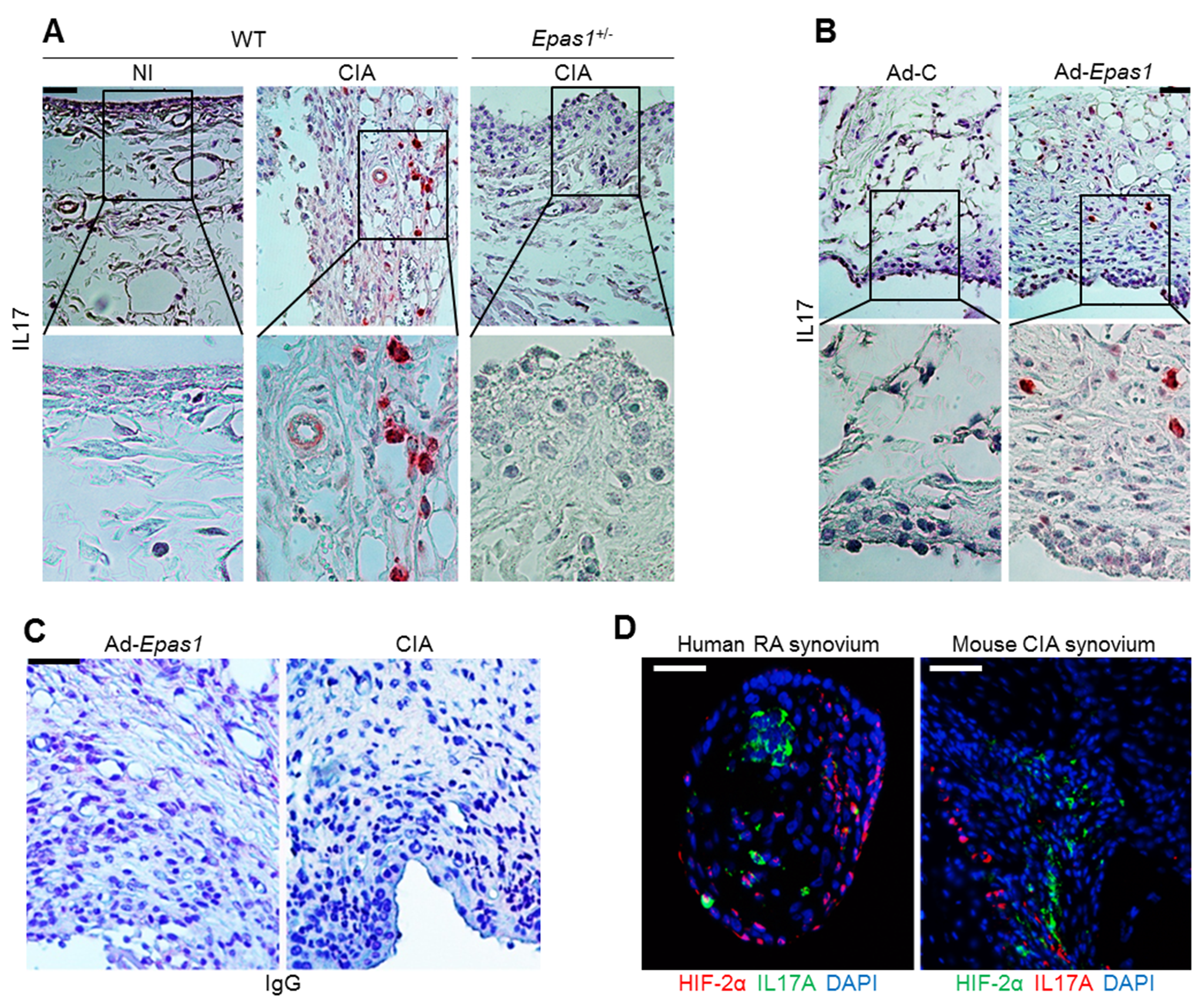

Supplement: Figure S5 — Epas1 knockdown in mice (Epas1 +/−) inhibits IL17 expression. (A) CIA was produced in WT and Epas1 +/− DBA/1J mice, and IL17 protein was detected in NI and CIA synovial sections after 6 wk by immunohistochemistry. (B) The knee joints of WT and Epas1 +/− DBA/1J mice were injected with Ad-C or Ad-Epas1 (1×10^9^ PFU). After 3 wk, synovial sections were immunostained for IL17 and counterstained with hematoxylin. (C) IgG was used as a negative immunostaining control. Representative images are shown (n = 6). (D) Typical images of triple-stained (HIF-2α, IL17A, and DAPI) human RA synovium and mouse CIA synovium (n = 6). Scale bar, 50 µm. (TIF) [file pbio.1001881.s005.tif]
