# Supplementary material for: Hypoxia-Inducible Factor-2α Is an Essential Catabolic Regulator of Inflammatory Rheumatoid Arthritis
Source: PLoS Biol. 2014 Jun 10;12(6):e1001881. doi: 10.1371/journal.pbio.1001881 (PMC4051611; doi:10.1371/journal.pbio.1001881)
Supplement: Table S1 — Clinical characteristics of RA patients with synovial biopsy. (DOCX) [file pbio.1001881.s006.docx]

**Table S1**. Clinical characteristics of RA patients with synovial biopsy*

| Assessment | RA (*n* = 10) |
| --- | --- |
| Age, median (range) | 54 (43-69) |
| Female, *n* (%) | 8 (80) |
| Disease duration (yr), median (range) | 6 (2-10) |
| Tender joint count, *n* (range) | 6 (4-10) |
| Swollen joint count, *n* (range) | 4 (2-8) |
| Patients global assessment (mm), median (range) | 60 (45-90) |
| ESR (mm/h), median (range) | 58 (35-110) |
| CRP (mg/dl), median (range) | 2.10 (1.02-3.24) |
| DAS 28-joint assessment, median (range) | 5.61 (4.63-7.11) |
| RF positivity, *n* (%) | 9 (90) |
| Anti-CCP antibody positivity, *n* (%) | 8 (80) |
| Current medication (within 3 mo) |  |
| Prednisolone (mg), median (range) | 7.5 (2.5-10) |
| Methotrexate, *n* (%) | 9 (90) |
| Sulfasalazine, *n* (%) | 8 (80) |
| Hydroxychloroquine, *n* (%) | 9 (90) |
| Leflunomide, *n* (%) | 1 (10) |
| Tacrolimus, *n* (%) | 1 (10) |
| Anti-TNF therapy, *n* (%) | 1 (10) |

*Patients who fulfilled the 2010 ACR/EULAR criteria for rheumatoid arthritis were included. Anti-CCP, anti-cyclic citrullinated peptide; CRP, C-reactive protein; DAS, disease activity score; ESR, erythrocyte sedimentation rate; n, number; RA, rheumatoid arthritis; RF, rheumatoid factor; TNF, tumor necrosis factor.
