# Supplementary material for: Hypoxia-Inducible Factor-2α Is an Essential Catabolic Regulator of Inflammatory Rheumatoid Arthritis
Source: PLoS Biol. 2014 Jun 10;12(6):e1001881. doi: 10.1371/journal.pbio.1001881 (PMC4051611; doi:10.1371/journal.pbio.1001881)
Supplement: Table S2 — Clinical characteristics of two patients with gouty arthritis. (DOCX) [file pbio.1001881.s007.docx]

**Table S2**. Clinical characteristics of two patients with gouty arthritis

| Assessment | Patient No. 1 | Patient No. 2 |
| --- | --- | --- |
| Age  Sex  Disease duration (yr)  Swollen joint count, *n*  Location of tophi  BMI (kg/m^2^)  CRP (mg/dl)  ESR (mm/h)  Serum urate (mg/dl)  Comorbidity  Hypertension  Diabetes  Dyslipidemia  Cardiovascular disease  Renal disease  Concomitant medication  Colchicine  NSAID  Allopurinol  Benzbromarone  Prednisolone  HIF-2α staining | 67  Male  30  10  Both 1^st^ MTP joints  Both lateral malleolus  23.4  4.3  62  9.6  +  -  -  -  +  +  -  +  -  -  Negative | 49  Male  7  4  Both 1^st^ MTP joints,  Both knee, right elbow joints  29.3  2.5  48  6.6  +  -  +  -  -  +  -  +  -  -  Negative |

BMI, body mass index; CRP, C-reactive protein; ESR, erythrocyte sedimentation rate; NSAID, non-steroidal anti-inflammatory drug; MTP, metatarsophalangeal joint; n, number.
