# Supplementary material for: Hypoxia-Inducible Factor-2α Is an Essential Catabolic Regulator of Inflammatory Rheumatoid Arthritis
Source: PLoS Biol. 2014 Jun 10;12(6):e1001881. doi: 10.1371/journal.pbio.1001881 (PMC4051611; doi:10.1371/journal.pbio.1001881)
Supplement: Table S3 — Clinical characteristics of two patients with psoriatic arthritis. (DOCX) [file pbio.1001881.s008.docx]

**Table S3**. Clinical characteristics of two patients with psoriatic arthritis.

| Assessment | Patient No. 1 | Patient No. 2 |
| --- | --- | --- |
| Sex  Age  Disease duration (yr)  Arthritic joint count, *n*  RF (IU/ml)  BMI (kg/m^2^)  ESR (mm/h)  CRP (mg/l)  Duration of skin disease (yr)  Duration of joint disease (yr)  Psoriasis area severity index  Visual analogue scale for pain (VAS)  CASPAR score (0-6)  Comorbidity  Hypertension  Diabetes  Dyslipidemia  Cardiovascular  Concomitant medication  NSAIDs  Prednisolone  Topical steroid  Methotrexate  Anti-TNF therapy  HIF-2α staining | Female  69  22  12  8.5 (negative)  33.29  14  2.4  22  18  40.8  9  5  +  -  +  -  +  +  +  -  -  Negative | Male  77  11  10  7.6 (negative)  22.03  7  0.6  11  8  19.5  7  4  +  +  -  -  +  +  +  -  -  Negative |

CASPAR, classification of psoriatic arthritis; CRP, C-reactive protein; ESR, erythrocyte sedimentation; RF, rheumatoid factor.
