# Supplementary material for: Hypoxia-Inducible Factor-2α Is an Essential Catabolic Regulator of Inflammatory Rheumatoid Arthritis
Source: PLoS Biol. 2014 Jun 10;12(6):e1001881. doi: 10.1371/journal.pbio.1001881 (PMC4051611; doi:10.1371/journal.pbio.1001881)
Supplement: Table S4 — PCR primers and conditions. (DOCX) [file pbio.1001881.s009.docx]

**Table S4**. PCR primers and conditions

| Gene | Origin | Strand | Sequence | Size  (bp) | AT^a^  (°C) |
| --- | --- | --- | --- | --- | --- |
| *Adamts4* | Mouse | ^b^S  ^c^As | 5'-CATCCGAAACCCTGTCAACTTG-3'  5'-GCCCATCATCTTCCACAATAGC-3' | 281 | 62 |
| *Adamts5* | Mouse | S  As | 5'-GCCATTGTAATAACCCTGCACC-3'  5'-TCAGTCCCATCCGTAACCTTTG-3' | 292 | 58 |
| *Ccl2* | Mouse | S  As | 5'-GGCCTGCTGTTCACAGTTGGC-3'  5'-GCTGAAGACCTTAGGGCAGATGCA-3' | 314 | 60 |
| *Ccl5* | Mouse | S  As | 5'-CTCACCATCATCCTCACTG-3'  5'-CTAGCTCATCTCCAAATAGTTG-3' | 255 | 60 |
| *Ccl7* | Mouse | S  As | 5'-GCTTTCAGCATCCAAGTGTG-3'  5'-TTCAGCACAGACTTCCATGC-3' | 180 | 60 |
| *Cxcl1* | Mouse | S  As | 5'-CGCCTATCGCCAATGAGCTG-3'  5'-CCAAGGGAGCTTCAGGGTCAAG-3' | 174 | 60 |
| *Cxcl2* | Mouse | S  As | 5'-AGTGAACTGCGCTGTCAATG-3'  5'-GCCTTGCCTTTGTTCAGTATC-3' | 203 | 60 |
| *Cxcl5* | Mouse | S  As | 5'-GCTGCGTTGTGTTTGCTTAACC-3'  5'-TCTTTCCACTGCGAGTGCATTC-3' | 232 | 60 |
| *Cxcl10* | Mouse | S  As | 5'-TTTCTGCCTCATCCTGCTG-3'  5'-GATGGTCTTAGATTCCGGATTC-3' | 223 | 60 |
| *Epas1* | Mouse | S  As | 5'-CGAGAAGAACGACGTGGTGTTC-3'  5'-GTGAAGGCGGGCAGGCTCC-3' | 370 | 63 |
| *Gapdh* | Mouse | S  As | 5'-TCACTGCCACCCAGAAGAC-3'  5'-TGTAGGCCATGAGGTCCAC-3' | 450 | 55 |
| *Hif1a* | Mouse | S  As | 5'-GCTGAAGACACAGAGGCAAA-3'  5'-ATACTTGGAGGGCTTGGAGA-3' | 333 | 60 |
| *Hprt* | Mouse | S  As | 5'-TTATGGACAGGACTGAAAGAC-3'  5'-GCTTTAATGTAATCCAGCAGGT-3' | 127 | 60 |
| *Il1b* | Mouse | S  As | 5'-TTGACAGTGATGAGAATGACC-3'  5'-GCAGGTTATCATCATCATCC-3' | 300 | 65 |
| *Il6* | Mouse | S  As | 5'-ACCACTCCCAACAGACCTGTCTATACC-3'  5'-CTCCTTCTGTGACTCCAGCTTATCTGTTAG-3' | 435 | 60 |
| *IL11* | Mouse | S  As | 5'-ATGAACTGTGTTTGTCGCCTGGTCCTG-3'  5'-CATCAAGAGCTGTAAACGGCGGAGTAG-3' | 432 | 60 |
| *Il12* | Mouse | S  As | 5'-ACGTTTATGTTGTAGAGGTGGACTGGACTC-3'  5'-ATACTTCTCATAGTCCCTTTGGTCCAGTG-3' | 476 | 60 |
| *Il17a* | Mouse | S  As | 5'-TTCATCTGTGTCTCTGATGCT-3'  5'- TTGACCTTCACATTCTGGAG -3' | 131 | 59 |
| *Il17f* | Mouse | S  As | 5'-CCCATGGGATTACAACATCACTC-3'  5'-CACTGGGCCTCAGCGATC-3' | 66 | 58 |
| *Il21* | Mouse | S  As | 5'-ATGGAGAGGACCCTTGTCTGTCTG-3'  5'-TATGTGCTTCTGTTTCTTTCCTCC-3' | 336 | 60 |
| *Lif* | Mouse | S  As | 5'-ATTGTGCCCTTACTGCTGCTGGTTCTG-3'  5'-GCCTGGACCACCACACTTATGACTTGC-3' | 584 | 60 |
| *Mmp2* | Mouse | S  As | 5'-CCAACTACGATGATGAC-3'  5'-ACCAGTGTCAGTATCAG-3' | 233 | 60 |
| *Mmp3* | Mouse | S  As | 5'-CTGTGTGTGGTTGTGTGCTCATCCTAC-3'  5'-GGCAAATCCGGTGTATAATTCACAATC-3' | 350 | 58 |
| *Mmp9* | Mouse | S  As | 5'-CGACCATACAGATACTG-3'  5'-ACCACATCGAACTTCGA-3' | 212 | 58 |
| *Mmp12* | Mouse | S  As | 5'-CCCAGAGGTCAAGATGGATG-3'  5'-GGCTCCATAGAGGGACTGAA-3' | 482 | 60 |
| *Mmp13* | Mouse | S  As | 5'-TGATGGACCTTCTGGTCTTCTGGC-3'  5'-CATCCACATGGTTGGGAAGTTCTG-3' | 473 | 58 |
| *Mmp14* | Mouse | S  As | 5'-GTGCCCTAGGCCTACATCCG-3'  5'-TTGGGTATCCATCCATCACT-3' | 580 | 55 |
| *Mmp15* | Mouse | S  As | 5'-GAGAGATGTTTGTGTTCAAGGG-3'  5'-TGTGTCAATGCGGTCATAGGG-3' | 260 | 55 |
| *Nos2* | Mouse | S  As | 5'-TCACTGGGACAGCACAGAAT-3'  5'-TGTGTCTGCAGATGTGCTGA-3' | 510 | 63 |
| *Pf4* | Mouse | S  As | 5'-GAGCGTCGCTGCGGTGTTTC-3'  5'-TAGGGGTGCTTGCCGGTCCAG-3' | 280 | 66 |
| *Ptgs2* | Mouse | S  As | 5'-GGTCTGGTGCCTGGTCTGATGAT-3'  5'-GTCCTTTCAAGGAGAATGGTGC-3' | 724 | 65 |
| *Tnfa* | Mouse | S  As | 5'-CTTGTCTACTCCCAGGTTCTCTTC-3'  5'-ACAGAGCAATGACTCCAAAGTAGACC-3' | 301 | 58 |
| *Tnfsfl11* | Mouse | S  As | 5'-GACTCGACTCTGGAGAGTGAAGAC-3'  5'-AATGTTGGCGTACAGGTAATAGAAG-3' | 354 | 63 |
| *Vegf* | Mouse | S  As | 5'-AGTCCCATGAAGTGATCAAGTTCA-3'  5'-ATCCGCATGATCTGCATGG-3' | 220 | 63 |

^a^AT, annealing temperature; ^b^S, sense primer; ^c^As, antisense primer
