# Supplementary material for: Hypoxia-Inducible Factor-2α Is an Essential Catabolic Regulator of Inflammatory Rheumatoid Arthritis
Source: PLoS Biol. 2014 Jun 10;12(6):e1001881. doi: 10.1371/journal.pbio.1001881 (PMC4051611; doi:10.1371/journal.pbio.1001881)
Supplement: Table S5 — siRNA sequences. (DOCX) [file pbio.1001881.s010.docx]

**Table S5.** siRNA sequences

| Species | Strand | Sequences |
| --- | --- | --- |
| Mouse *Epas1* | Sense  Antisense | 5'-CUCAGUUACAGCCACAUCGUCACUG-3'  5'-CAGUGACGAUGUGGCUGUAACUGAG-3' |
| Mouse *Epas1* | Sense  Antisense | 5'- GGAGAUGCCAUUAUUUCUCUCGAUU -3'  5'- AAUCGAGAGAAAUAAUGGCAUCUCC-3' |
| Control siRNA | Sense  Antisense | 5'-CCUACGCCACCAAUUUCGU-3'  5'-ACGAAAUUGGUGGCGUAGG-3' |
